# Crystal‐Size‐Induced Band Gap Tuning in Perovskite Films

**DOI:** 10.1002/anie.202106394

**Published:** 2021-08-18

**Authors:** Amita Ummadisingu, Simone Meloni, Alessandro Mattoni, Wolfgang Tress, Michael Grätzel

**Affiliations:** ^1^ Laboratory of Photonics and Interfaces (LPI) Institute of Chemical Sciences and Engineering École Polytechnique Fédérale de Lausanne (EPFL) Station 6 1015 Lausanne Switzerland; ^2^ Dipartimento di Ingegneria Meccanica e Aerospaziale Università di Roma “Sapienza” via Eudossiana 19 00184 Roma Italy; ^3^ Dipartimento di Scienze Chimiche Farmaceutiche e Agrarie (DOCPAS) Università degli Studi di Ferrara (Unife) Via Luigi Borsari 46 44121 Ferrara Italy; ^4^ Istituto Officina dei Materiali IOM—CNR Consiglio Nazionale delle Ricerche, Cagliari Cittadella Universitaria 09042 Monserrato (Ca) Italy; ^5^ Laboratory of Photomolecular Science (LSPM) Institute of Chemical Sciences and Engineering École Polytechnique Fédérale de Lausanne (EPFL) Station 6 1015 Lausanne Switzerland

**Keywords:** band gap, perovskites, photoluminescence, quantum confinement, solar cells

## Abstract

A comprehensive picture explaining the effect of the crystal size in metal halide perovskite films on their opto‐electronic characteristics is currently lacking. We report that perovskite nanocrystallites exhibit a wider band gap due to concurrent quantum confinement and size dependent structural effects, with the latter being remarkably distinct and attributed to the perturbation from the surface of the nanocrystallites affecting the structure of their core. This phenomenon might assist in the photo‐induced charge separation within the perovskite in devices employing mesoporous layers as they restrict the size of nanocrystallites present in them. We demonstrate that the crystal size effect is widely applicable as it is ubiquitous in different compositions and deposition methods employed in the fabrication of state‐of‐the‐art perovskite solar cells. This effect is a convenient and effective way to tune the band gap of perovskites.

## Graphical abstract

Energy levels of perovskite nanocrystallites depend on their size owing to quantum confinement and the concurrent yet distinct phenomenon of size‐dependent structural changes where surface perturbations template the core. This effect can aid in the design of perovskite opto‐electronic devices utilizing mesoporous layers to tailor the size of infiltrated nanocrystallites.

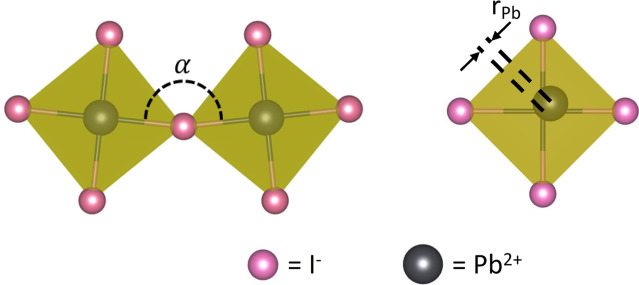

## Introduction

Perovskite materials have recently emerged as excellent candidates for opto‐electronic applications, photovoltaics in particular. Several perovskite deposition methods have been developed in the recent past for the fabrication of prototype perovskite solar cells. The most commonly used ones are the sequential deposition method^[1]^ and the anti‐solvent method.^[2]^ The former involves two steps, the deposition of the lead iodide (PbI_2_) as a film and the dipping of the film in a methylammonium iodide (CH_3_NH_3_I, MAI) solution to form the perovskite, methylammonium lead iodide (CH_3_NH_3_PbI_3_, MAPbI_3_) in‐situ.^[1]^ The latter involves the deposition of a perovskite precursor solution containing the reactants needed to form the perovskite, followed by dripping of an anti‐solvent on top and heating to form the perovskite thin film.^[2]^

Perovskite solar cells have also been developed in a variety of architectures, including the mesoscopic,^[2]^ planar^[3]^ and inverted ones.^[4]^ The state‐of‐the‐art mesoscopic cells^[2]^ are based on a mesoporous layer of TiO_2_ particles, which serves as the electron transport layer. Such cells consist of a capping layer of perovskite crystals on top of the mesoscopic TiO_2_ layer, which is itself infiltrated with perovskite. In contrast, planar architectures consist only of a compact perovskite layer between electron and hole transport layer,^[5]^ with the simpler architecture presenting its own advantages.

Reports^[6]^ describe spectroscopic investigations looking into the correlation between perovskite microstructure and key opto‐electronic properties. They identified that properties such as the optical absorption,[^6b^, ^6c^] the photoluminescence^[6c]^ (PL) and the electron‐hole interaction,^[6a]^ which are important for photovoltaics, are dependent on whether the perovskite lies in the mesoscopic or capping layer. Specifically, the main differentiating aspect of the microstructure in these two cases: the crystal size, has been of interest in the past. While several aspects commonly associated with the crystal size such as lattice strain have been proposed as possible explanations for the variations in opto‐electronic properties observed when crystal size changes, the underlying cause remains elusive.[^6a^, ^6b^] In order to effectively design and fabricate solar cells to achieve the highest performances, knowledge about the influence of crystal size on some of the relevant material properties is essential. Thus, a fundamental explanation behind this phenomenon needs to be identified and this is the focus of this study.

Herein, we aim to address the above‐mentioned aspects by coupling experimental observations with insights from classical and first principle atomistic simulations. First, we investigate the steady state PL from methylammonium lead iodide perovskite (CH_3_NH_3_PbI_3_) samples at different stages of conversion from lead iodide (PbI_2_) in the sequential deposition reaction^[1]^ and observe a blue‐shift of the PL spectra and an unusual asymmetry in the emission weighted towards higher energies at the start of the reaction. Then, from classical molecular dynamics (MD) and density functional theory (DFT) calculations, we identify that the band gap narrows with increase in perovskite nanocrystallite size, independent of quantum confinement (QC). This is because PbI_6_ octahedra in smaller nanocrystallites are more tilted and Pb atoms are more off‐centered. We associate this finding with the presence of smaller sized nanocrystallites at the start of the reaction, which manifests itself as a blue‐shift and an asymmetry in the emission at the early stages of the reaction.

Next, using cross‐sectional confocal laser scanning fluorescence microscopy (CLSM), we show that the CH_3_NH_3_PbI_3_ perovskite in the mesoporous layer has a blue‐shifted emission compared to the capping layer. We also examine perovskite samples of the nominal composition Cs_0.05_MA_0.16_FA_0.79_Pb(I_0.83_Br_0.17_)_3_ made using the anti‐solvent method and show that the crystal size effect is present in these as well, demonstrating the broad implications of our work. Examining samples made on mesoscopic scaffolds of different particle sizes, which restrict the size of perovskite crystallites in them, we clearly show that crystal size can be used to tune opto‐electronic properties such as the band gap. Finally, we discuss the direct implications of our observations for the performance of mesoporous layer‐based devices and propose the use of crystal‐size‐control achieved via tailoring of the mesoporous scaffold for effective photovoltaic device design.

## Results and Discussion

The sequential deposition method used to deposit perovskite thin films is interesting for kinetic studies as it allows us to accurately follow and monitor the progress of the perovskite formation. The concentration of the MAI solution can be kept low enough to practically halt the reaction. This allows us to control the progress of the reaction, thus monitoring the changes in the film, such as the growth of perovskite nanocrystallites, as the reaction progresses. This makes the sequential deposition method an apt choice for this part of the study rather than the anti‐solvent method. Furthermore, the sequential deposition has been demonstrated as a viable option for the large‐scale production of perovskite thin films for opto‐electronics through scaling up of the process.^[7]^ This exemplifies the great potential of this method and the need to study it further.

Following the sequential deposition method here, PbI_2_ films are deposited onto a mesoporous Al_2_O_3_ scaffold and then dipped in a MAI solution in 2‐propanol to form a CH_3_NH_3_PbI_3_ film (see the Supporting Information, Materials and Methods). The path of this reaction has been reported in our previous study.^[8]^ Al_2_O_3_ scaffolds rather than TiO_2_ were used for these measurements to prevent quenching of the PL signal. To begin with, we look at the steady‐state PL spectra of samples made using sequential deposition where the reaction was arrested at different points of conversion from PbI_2_ to CH_3_NH_3_PbI_3_ (see ex‐situ measurements in the Supporting Information for details).

**Figure 1:**
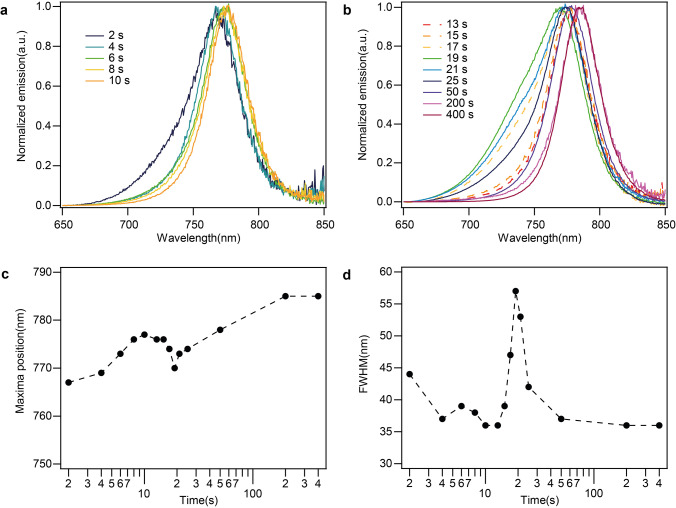
Photoluminescence spectra of samples of different dipping times in sequential deposition. a),b) Normalized emission spectra of samples of gradually increasing dipping times of 2, 4, 6, 8, 10, 13, 15, 17, 19, 21, 25, 50, 200 and 400 s. c) Maxima position and d) FWHM obtained from each of the spectra shown in (a) and (b).

The emission spectra of these PbI_2_ samples dipped in MAI solution for different periods (dipping time), containing different quantities of CH_3_NH_3_PbI_3_ and unconverted PbI_2_, are presented in Figure 1 a and b. We assign the emission between 700 and 800 nm to CH_3_NH_3_PbI_3_ at room temperature based on the literature.^[6c]^ Interestingly, we observe that the CH_3_NH_3_PbI_3_ emission spectra unexpectedly shift to higher wavelengths as dipping time increases, i.e., as the conversion reaction from PbI_2_ to CH_3_NH_3_PbI_3_ progresses. Through further analysis of these spectra (discussed in the SI), we identify that a reduction of the full width at half maximum (FWHM) (Figure 1 d) and the high energy tail in the emission takes place with time. The emission from unreacted PbI_2_ is expected to be between 500 and 550 nm and therefore does not interfere with the emission detected from the perovskite.^[8]^

Complementary measurements (see SI, Figure S1) demonstrate that the PL shift is also detected in in‐situ measurements where the sequential deposition reaction have not been arrested at different points in time. Through another investigation (Figure S2) of samples with crystals in the capping layer of increasing sizes deposited on identical mesoporous scaffolds, we observe that smaller crystals in the capping layer also exhibit blue‐shifted emission compared to larger crystals. The phenomenon we report is therefore not limited to perovskite nanocrystallites in the mesoporous layer, but also applies to crystals in the capping layer. This finding is also consistent with the literature investigating mesoscopic and planar films deposited separately.[^6a^, ^6c^]

According to the literature, the formation of the perovskite from the PbI_2_ takes place via the intercalation of MAI into the PbI_2_ followed by structural reorganization of the crystal lattice. In this stage, perovskite formation in the capping layer proceeds through an increase in the size of the perovskite nanocrystallites in the PbI_2_ precursor with dipping time.^[8]^

In this work we consider two hypotheses: i) that the red‐shifting with dipping time is due to either the reduction of QC or ii) to structural changes with the growth of the perovskite nuclei. Previous reports have considered QC mainly focusing on the particle‐in‐a‐box effect,^[9]^ which widens the band gap in smaller nuclei. Here we also consider the effect of enhanced exciton binding associated to the confinement, which has the opposite effect on the band gap.

The reduction in QC alone cannot explain the red‐shift of the PL peak with dipping time. In fact, we still observe a significant shift when nuclei largely exceed Bohr exciton radius of the perovskite (*a*_0_=2.2 nm),^[10]^ as indicated by the detection of the X‐ray diffraction (XRD) signal for dipping time as short as 27 s, implying that the film contains nuclei larger than 10 nm (Figure S3), the approximate detection limit of our XRD measurements. This aspect is explored further at a later point.

Reports[^6c^, ^11^] have pointed to the possibility that the perovskite band gap is size dependent, with smaller crystals exhibiting larger band gaps as a consequence of lattice strain. To begin with, Grancini et al.^[6c]^ posited that an ordered arrangement of the organic cations within the inorganic cage in larger crystallites grown on a flat substrate results in a reduction in the strain felt on the Pb‐I cage when compared to smaller ones grown in the mesoporous scaffold. This in turn red‐shifted the optical absorption onset. However, as we show later, we observe no change in the orientation of cations with grain size. Drawing from the conclusions of the above report^[6c]^ among others, D'Innocenzo et al.^[11]^ in turn suggested that the reduction in the band gap for larger crystals is possibly due to a change in the stress in the Pb−I bond. A precise identification of the nature of this stress and a quantification of the effect on the band gap is still lacking.

Surface effects, limited to the context of those resulting in structural strains and doping^[6b]^ or dislocations,^[12]^ have been suggested as the possible origin of the red‐shift in the emission with crystal size. Nevertheless, the surface‐induced structural effects in perovskite crystals have not yet been fully studied or understood. Here, we perform simulations that will identify the origin of the red‐shift and the reduction in the asymmetry of the emission observed with increasing dipping time, paying close attention to surface‐induced effects.

To identify the origin of the change in emission (red‐shift and decrease in the FWHM) with dipping time, we perform combined classical MD and DFT simulations of clusters of increasing size modeling perovskite nanocrystallites forming and growing in the PbI_2_ precursor. Classical MD allows us to analyze relatively large nanocrystallites investigating the effect of their size on the structure. The classical model potential for hybrid perovskites (MYP) developed by Mattoni et al.^[13]^ is used here. MYP has been shown to give excellent results in predicting structural and dynamical bulk^[14]^ and non‐bulk properties of halide perovskites,^[15]^ including surfaces[^15a^, ^16^] and grain boundaries.^[17]^ See Materials and Methods for additional details and references.

**Figure 2:**
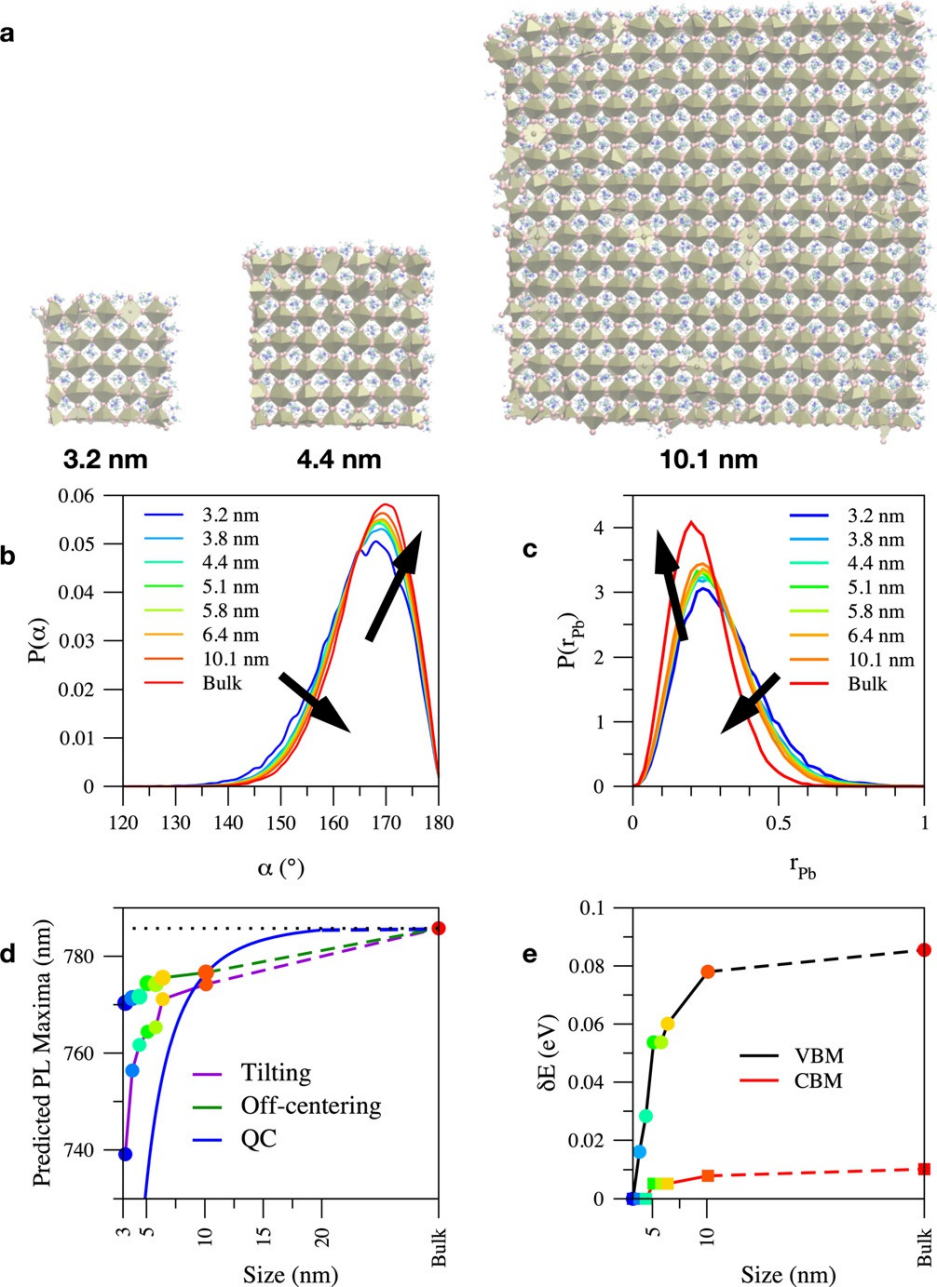
Results of atomistic simulations on CH_3_NH_3_PbI_3_ nanocrystallites of increasing size. a) Snapshots of the 3.2, 4.4 and 10.1 nm nanocrystallites taken from the classical MD. b) Pb‐I‐Pb angular distribution function, P(*α*) c) off‐centering distribution, P(*r*_Pb_). The maximum of P(*r*_Pb_) is at *r*_off_≈0.2 nm, consistent with literature data on the CsSnBr_3_ analogue.^[21]^ d) Estimated wavelength of the PL peak as a function of the nanocrystallite size due to Pb‐I‐Pb distortions, off‐centering and quantum confinement. Theoretical values have been rigidly shifted to meet the bulk value. e) Energy of the valence band maximum (VBM) and conduction band minimum (CBM) as a function of the nanocrystallite size due to Pb‐I‐Pb distortion. Data are relative to the smallest nanocrystallite, *δE*=*E*(l)−*E*(3.2 nm). For reference, we also report data for an ideal (infinite) CH_3_NH_3_PbI_3_ crystal. The colors of the symbols in (d) and (e) denote the sizes of the nanocrystallites according to the colors in the legend in (b). We remark that the difference between the band gap of a ca. 10 nm nanocrystallite and the bulk crystal due to surface‐induced structural effects corresponds to a shift in emission of ca. 13 nm, while the difference due to QC is ca. 9 nm. We also look at the apparent abnormal trend of the predicted PL maximum of the “tilting” curve (in (d)) and VBM curve (in (e)) between clusters of 5.1 and 5.8 nm. This is likely a result of our classical MD+DFT simulation strategy that, while allowing to treat large clusters, is affected by statistical fluctuations, resulting in (random) errors like in experiments. More details are available in the SI.

The growing nanocrystallites of CH_3_NH_3_PbI_3_ are modeled as cubic‐like clusters ranging from ca. 3 to ca. 10 nm (Figure 2 a). This shape is consistent with the TEM images of nanocrystals in the literature^[18]^ and classical MD and DFT results showing that the (100) surface is the most stable one.[^15a^, ^19^] Additional details on the model chosen to represent CH_3_NH_3_PbI_3_ nanocrystals are discussed in the Materials and Methods section, where all the computational details are also provided. This model takes into account both local (e.g., surface defects) and global (e.g., Laplace pressure) effects of the nanocrystallite surface. For reference, we also consider a CH_3_NH_3_PbI_3_ (infinite periodic) bulk sample.

**Figure 3:**
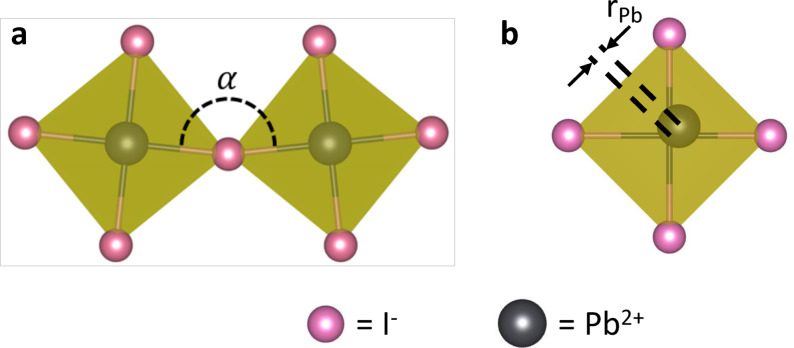
Visual representation of PbI_6_ octahedra in CH_3_NH_3_PbI_3_ nanocrystallites showing the two structural characteristics affecting the electronic structure of perovskite. a) Definition of the Pb‐I‐Pb angle *α. α* is a proxy of the tilting angle. In configurations extracted from MD simulations, in which PbI_6_ octahedra are dynamically distorted, *α* is easier to compute than the tilting angle. b) Off‐centering is defined as the distance between the barycenter of the iodine atoms of a PbI_6_ octahedron and the corresponding lead atom.

Four structural characteristics affecting the electronic structure of perovskite, the Pb‐I bond length^[20]^ (Figure S4), the Pb‐I‐Pb angle (Figure 2 b), the off‐centering of Pb atoms in the PbI_6_ octahedron^[21]^ (Figure 2 c) and the reorientation of the cation^[6c]^ (Figure S5) are considered. The Pb‐I bond length does not show any significant dependence or clear trend with nanocrystallite size (as shown in Figure S4). Similarly, the distribution of the orientation of cations is not affected by the nanocrystallite size (Figure S5). The Pb‐I‐Pb angle (*α*) is a convenient measure of the tilting of PbI_6_ octahedra,^[22]^ the former is easier to compute than the latter in MD, in which PbI_6_ octahedra are dynamically distorted. A visual representation of the angle *α* between adjacent PbI_6_ octahedra is shown in Figure 3 a. P(*α*) is characterized by an intense peak at ca. 170° corresponding to the tilted quasi‐cubic structure of halide perovskites (Figure 2 b). We also find Pb‐I‐Pb angles close to 90° but these are associated with defects forming at the surfaces, edges and corners of nanocrystallites.

The P(*α*) becomes sharper and shifts towards higher angles with increase in nanocrystallite size (Figure 2 b). The P(*α*) is broader near the surface of the nanocrystallite than in the bulk and the reduced fraction of stoichiometric units in the former region with respect to the latter in larger nanocrystallites explains the sharpening of *α* with their size. This can be explained considering that the larger core, exhibiting a more bulk‐like crystalline order, has a templating effect on the periphery, making the structure of this region more regular (Figure S6). Since the crystal size increases with dipping time, we associate the effects of the nanocrystallite size on the Pb‐I‐Pb distribution with dipping time.

The off‐centering distribution of Pb from the nominal center of the PbI_6_ octahedron (Figure 3 b), measured by the average of the iodine positions, P(*r*_Pb_), is shown in Figure 2 c for nanocrystallites of various sizes. We see that the maximum of P(*r*_Pb_) shifts to slightly shorter distances and the distribution becomes sharper with nanocrystallite size. Similar to the *α* case, P(*r*_Pb_) is broader at the periphery of the nanocrystallite than in the core and since the core/periphery ratio increases with nanocrystallite size, we observe a templating effect in this case as well.

To understand the effect of changes of the Pb‐I‐Pb angle and Pb off‐centering on the PL spectrum as opposed to just QC, we perform DFT calculations of a bulk system using the *α*_max_ and *r*_Pb_^max^ values determined in the above‐described MD simulations (Figure S7).

Previous DFT calculations on small nanocrystallites have investigated the dependence of the band gap on their size^[23]^ without distinguishing between structural effects and QC. We find that a narrower band gap is associated with a larger *α*_max_, i.e., less tilted structures, and lower *r*_Pb_^max^, i.e., lower Pb off‐centering. Thus, simulations predict that surface‐induced structural effects produce a shift in the PL spectra of ca. 13 nm towards longer wavelengths between a ca. 10 nm nanocrystallite and the bulk (Figure 2 d) as an effect of the increase in *α*_max_ and a corresponding shift of ca. 8 nm due to the reduction of *r*_Pb_^max^, in good agreement with the experimental results from the steady state PL measurements presented earlier (Figure 1 c).

The narrowing of the band gap with increasing *α*_max_ and decreasing *r*_Pb_^max^ is due to the character of the valence band maximum (VBM) and the conduction band minimum (CBM) of CH_3_NH_3_PbI_3_, which are made of Pb‐6s/I‐5p and Pb‐6p/I‐5s antibonding atomic orbitals. The overlap of both orbitals increases with a larger *α*_max_ and a smaller *r*_Pb_^max^, and the VBM and CBM move towards the vacuum level.^[24]^ However, the change in the antibonding overlap of the VBM is stronger and the band gap narrows with the nanocrystallite size (Figure 2 e).

The other effect observed in the experimental steady‐state PL spectra, the decrease in the FWHM (Figure 1 d) and asymmetry, can also be attributed to the growth of perovskite crystallites. In the early stages of the reaction small perovskite crystals are formed, which grow larger at longer dipping times through the phenomenon of Ostwald ripening.^[8]^ Since the emission wavelength converges to the bulk value for larger crystallites, the shorter wavelength contribution of smaller crystallites disappears from the PL spectra, reducing the FWHM and asymmetry. We also observe an intrinsic sharpening and reduction of asymmetry in P(*α*) and P(*r*_Pb_) with nanocrystallite size (Figure 2 b and c), which is expected to result in a corresponding sharpening and symmetrization of the PL spectra with dipping time.

For a comprehensive image of the system, we look into QC effects for nanocrystallites of the same size considered above. The blue‐shift of the PL spectrum due to QC is the result of two terms of opposite sign: Firstly, the particle‐in‐a‐box effect, which widens the band gap and this widening is proportional to the inverse of the square size of the nanocrystallite and of the reduced mass of the exciton. Secondly, the enhanced exciton binding associated with the confinement of the corresponding electron and hole, which reduces the band gap and is proportional to the inverse of the size of the crystal^[25]^ (see Materials and Methods for details). This second contribution, which reduces the length scale of QC effect, has been often neglected in the literature.^[9]^ Here, we use the reduced mass of the exciton obtained from DFT data available in the literature,^[26]^ which is consistent with experimental values (0.15 m_0_).^[10]^ For very small nanocrystallites, the QC effect dominates. However, this effect vanishes rapidly thereafter and for larger nanocrystallites of ca. 10 nm, the surface‐induced structural effects (bending of the Pb‐I‐Pb angle and off‐centering of Pb) are larger than the QC, with the predicted shift in PL maxima obtained as ca. 13 and ca. 8 nm for the former vs. ca. 9 nm for the latter as shown in Figure 2 d. We see that QC becomes negligible for nanocrystallites of 15 nm (see Figure 2 d).

Summarizing the calculations, we show that the band gap narrows with the increase of nanocrystallite size due to surface‐induced structural effects, in particular a reduction in the tilting of PbI_6_ octahedra and off‐centering of Pb (as visually depicted in Figure 3 b). Thus, DFT calculations predict that the emission maxima red‐shifts with increase in the nanocrystallite size and hence with dipping time. However, for large nanocrystallites these effects become negligible, which is consistent with the observation that there is no change in the emission maxima between 200 and 400 s (Figure 1 c). We remark that due to these concomitant structural effects, QC alone is insufficient to explain the dependence of PL spectra on crystal size.

Our observations are in keeping with the “soft” nature of perovskite crystals, which has been demonstrated clearly by a number of recent studies. These revealed the unique feature of perovskites that tuning of the surface can affect the property evolution within the interior of perovskite crystals. Xue et al.^[27]^ have shown the occurrence of surface‐induced secondary grain growth in perovskite thin films, while we have recently reported on the long‐term stabilization of α‐FAPbI_3_ films by covering them with an overlayer of two‐dimensional perovskite.^[28]^ The soft nature of perovskites is responsible for the failure of the Fröhlich polaron model of electron‐lattice coupling to explain charge carrier transport,^[29]^ the formation and/or ordering of polar nanodomains surrounding charge carriers,^[30]^ and the simultaneous presence of charge carriers with different lifetimes.^[31]^ We believe that the soft nature of perovskites, which can react to “random” stress associated with different kinds of grain boundaries in polycrystalline films presenting non‐uniform strain, might be responsible for macroscopic inhomogeneity of optical (e.g., PL) characteristics of samples.^[32]^ Similarly, stress/strain induced by the interface with the substrate or layers underneath the perovskite, e.g., mesoscopic TiO_2_, might affect its local opto‐electronic properties.

**Figure 4:**
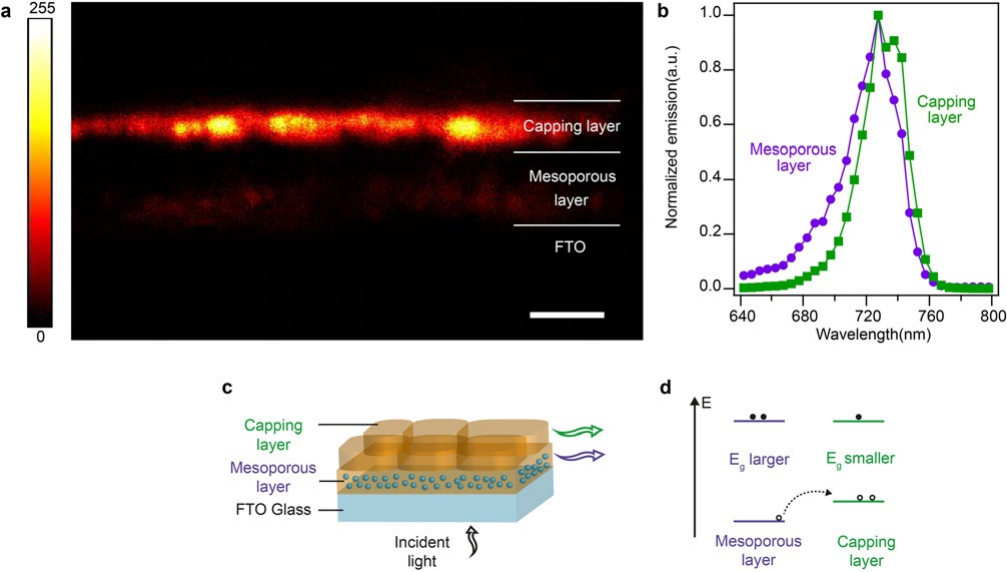
Study of the emission from the mesoporous layer and the capping layer in a partial cell. a) Cross‐sectional CLSM imaging of CH_3_NH_3_PbI_3_ sample consisting of a capping layer and the mesoporous perovskite infiltrated into an Al_2_O_3_ mesoporous scaffold. Pseudo‐color cross‐sectional image of fully converted sample. 32 images showing the emission between 640 and 800 nm in steps of 5 nm each have been summed. Color scale assignment to the emission intensity in the image shown. Scale bar, 2.5 μm. b) Normalized emission from the perovskite in the capping layer and the mesoporous layer obtained from the images that make up (a). The emission from the mesoporous layer is blue‐shifted compared to the capping layer. c) A view of a perovskite solar cell without a back contact. The emissions from the perovskite in the mesoporous layer and the capping layer in the partial cell are shown, d) energy band diagram depicting the perovskite levels in the mesoporous and capping layers.

We employ hyperspectral cross‐sectional CLSM to look at fully formed methylammonium lead iodide perovskite films on an Al_2_O_3_ mesoscopic scaffold to study the emission from the mesoporous and capping layers individually. The cross‐sectional imaging technique has been described in our previous work^[8]^ and in the Materials and Methods section. Figure 4 b shows the normalized emission spectra for perovskite in the mesoporous and the capping layers, where each data point was obtained from the images constituting Figure 4 a by isolating both the layers and averaging over the respective areas through image processing for each image (See Materials and Methods for details about sampling). We observe that there is a blue‐shift in the emission profile of the perovskite in the mesoporous layer. This observation is consistent with steady state PL measurements reported in the literature where perovskite infiltrated into a mesoscopic layer and deposited as a flat layer were compared. Perovskite infiltrated into the mesoporous scaffold consists of smaller crystals compared to the capping layer.^[6c]^ Therefore, based on our simulations, perovskite in mesoporous layers is expected to exhibit a blue‐shifted emission and our experimental findings confirm the same.

TiO_2_ mesoporous layers are employed in the fabrication of perovskite solar cells with mesoporous architectures.^[2]^ We characterize both the 18 NRT TiO_2_ and 30 NRD TiO_2_ mesoporous layers, those commonly used in solar cells, using scanning electron microscopy (Figure S8) and N_2_ gas adsorption measurements (Table S1). Using the Brunauer–Emmett–Teller (BET) method,^[33]^ we find that the average pore diameter of the mesoporous matrix is 26 and 31 nm for the 18 NRT and 30 NRD TiO_2_ mesoporous films, respectively. From the Barrett–Joyner–Halenda (BJH) method,^[33]^ the average pore diameter was found to be 22 and 27 nm for the 18 NRT and 30 NRD TiO_2_ mesoporous films, respectively.

The mesoporous scaffold restricts the size of perovskite crystals that can grow in its pores, as mentioned in the literature.^[34]^ Therefore, these perovskite crystals formed in‐situ in these mesoporous layers are likely to have a crystal size distribution with average diameters similar to those estimated for the average pore diameters of these mesoporous layers. The larger values for the average diameters when compared to the Bohr exciton radius again indicate that the QC regime has been surpassed for a large fraction of the crystals in the mesoporous layers as well. Furthermore, similar to the Al_2_O_3_ case imaged earlier (which consisted of mesoporous particles of comparable size), we expect the blue‐shift due to surface‐induced structural changes described earlier to take place for perovskite in TiO_2_ mesoporous layers as well. We expect this blue‐shifting to have no drawback on re‐absorption (see Sec. 6 in SI).

It is interesting to look at the schematic of a partial solar cell shown in Figure 4 c and the corresponding schematic of the energy band diagrams shown in Figure 4 d (see also Figure S9). Due to the predominance of small crystals in the mesoporous layer, the VBM is expected to be at more negative values compared to the capping layer (Figure 4 d) as identified thorough the above simulations. This offset in the VBMs of the perovskite in these layers has implications for the performance of mesoscopic solar cells as it can affect charge transport in the device. We posit that it provides additional selectivity for hole transport as photo‐generated holes in the mesoporous layer can be transported into the capping layer (as shown in Figure 4 d) where they come in contact with the hole transport material, while hole transport in the opposite direction is not favorable. Moreover, the reduction in the population of photo‐generated holes in the mesoporous layer compared to electrons lowers the possibility of recombination taking place there. The nearly unchanged CBM position in the perovskite (as shown in Figure 2 e) is an advantage as the alignment with the CBM of the TiO_2_ electron transport layer^[5]^ is maintained.

We also consider the disadvantage of mesoscopic architectures, the numerous grain boundaries in the perovskite in the mesoporous layer are likely to be potential recombination centers, which may result in performance losses, due to non‐radiative recombination lowering the open‐circuit voltage.^[35]^ However, our previous study^[2]^ with mesoporous‐based solar cells (composed of a Rb‐incorporated quadruple‐cation perovskite on ca. 100 nm thick TiO_2_ mesoporous layer) demonstrated open‐circuit voltages approaching theoretically achievable values, pointing to low losses due to non‐radiative recombination. While extremely thick mesoporous layers might adversely affect photovoltaic performance due to large losses from non‐radiative recombination, the use of thin mesoporous scaffolds still exploits the advantages discussed above.

Based on our findings, we propose a novel strategy wherein the mesoporous scaffold itself can be used to tune the VBM and CBM in devices and employed in designing new architectures for perovskite photovoltaics, especially band gap graded ones. This is an alternative to current design strategies such as compositional tuning where the perovskite composition would be graded through the depth of the device and would practically require an extremely well‐controlled reactant evaporation process for the deposition of the graded perovskite layer. Using mesoporous scaffolds of increasing sizes, we suggest utilizing the crystal‐size‐induced structural effects in each layer to tune the VBM and CBM (shown schematically in Figure S9 with details in the SI), to achieve a fully graded band gap with just one absorber material, fabricated using a convenient solution‐based deposition process. Experimental results relevant to this discussion on the opto‐electronic properties of perovskite in mesoporous scaffolds of different sizes are presented later.

Our results are particularly relevant for cells that already consist of multiple mesoporous layers such as those reported by Mei et al.^[36]^ This architecture is well suited to produce a graded band gap solar cell using several different mesoporous layers. Moreover, many of the highest performing prototype solar cells make use of mesoporous layers. These include those reported by Saliba et al.^[2]^ at 21.6 %, Tavakoli et al.^[37]^ at about 22 % and Min et al. over 23 %,^[38]^ which all employ 100–150 nm thick TiO_2_ mesoscopic layers. The same holds for perovskite devices showing the present certified record efficiency of 25.2 %.^[39]^ The perovskite in these mesoporous layers will likely be subject to the size effects explored in our study. Furthermore, the potential of mesoscopic TiO_2_ based perovskite solar cells fabricated with high efficiencies recently through inkjet printing has been demonstrated by Huckaba et al.^[40]^ which shows the scalability of this technology.

To explore the broader implications of our work, we examine perovskite samples of the nominal composition Cs_0.05_MA_0.16_FA_0.79_Pb(I_0.83_Br_0.17_)_3_, henceforth referred to as the triple‐cation composition, which is used in a number of high‐performing solar cells[^37^, ^41^] (Figure S10). This perovskite is predominantly composed mainly of formamidinium ions (FA, HC(NH_2_)_2_^+^) in place of methylammonium ions (MA, CH_3_NH_3_^+^). Moreover, the perovskite is deposited using the anti‐solvent method as described in the literature,^[41]^ which is the other deposition method widely used to deposit perovskite films for high‐performing devices such as those reported in these references.[^37^, ^41^] We perform hyperspectral cross‐sectional CLSM to study the emission from the perovskite in both the mesoporous layer and the capping layer in partial solar cells, in a manner similar to that done for the methylammonium lead iodide sample in Figure 4. We image two triple‐cation perovskite samples, one deposited on a mesoscopic scaffold made of 17 nm diameter Al_2_O_3_ particles (Figure S10a and b), while the other is made of 95 nm diameter Al_2_O_3_ particles (Figure S10c and d). The perovskite is infiltrated into the mesoporous layer and also forms an additional capping layer on top in both samples (Figure S11, discussed more later).

For both these samples, the normalized emission obtained from the images (Figure S10b and d) shows that the emission maxima of the perovskite in the mesoporous layer is blue‐shifted compared to the capping layer, similar to observations made for methylammonium lead iodide in Figure 4. Moreover, we observe longer tails in the emissions towards the blue (high energy, associated with larger band gaps) for the perovskite in the mesoporous layers compared to the capping layers, which we attribute to the size restriction of nanocrystallites in the mesoporous layers. This is comparable to our previous investigation of methylammonium lead iodide samples made with short dipping times in the sequential deposition method, where small sized nanocrystallites are expected to form. These findings are therefore consistent with our experiments and theoretical results discussed earlier in this report. In more general terms, we demonstrate that the crystal size effect we have described above for methylammonium lead iodide is also clearly observed for this complex triple‐cation composition deposited via the anti‐solvent method.

Comparing the samples with each other (Figure S10b and d), we notice that perovskite nanocrystallites in both the mesoporous layers have different band gaps and so do the capping layers. We see that the CLSM spectra from the perovskite in the mesoporous and capping layers from the 95 nm sample (Figure S10d) exhibit emission maxima at longer wavelengths, associated with smaller band gaps, when compared to the corresponding spectra from the 17 nm sample (Figure S10b). We take a closer look at the morphology of the perovskite in the mesoporous and capping layers for both samples using scanning electron microscopy (SEM) (Figure S11). The size of perovskite crystals in the 95 nm sample is larger in both the capping and mesoporous layers compared to the 17 nm sample in these SEM images. There are two implications for these observations:

Firstly, the smaller band gap of the perovskite in the mesoporous layer associated with the lower energy emission from the 95 nm mesoporous layer sample, compared to the one from the 17 nm sample, supports our previous hypothesis. For crystallites of larger size obtained within the 95 nm mesoporous scaffold, which is well beyond the QC regime (ca. 20 times the Bohr exciton diameter: d_0_ of methylammonium lead iodide perovskite,^[10]^ ca. 15 times d_0_ of FAPbBr_3_ and ca. 7 times d_0_ of FAPbI_3_,^[42]^ which we take as a reference for the unknown value of the Bohr exciton radius of mixed perovskites), the crystal size effect is present and it is an effect distinct from QC. For very small nanocrystallites, this crystal size effect occurs in addition to and alongside QC. Therefore, comparing the CLSM results for the 17 nm and 95 nm mesoporous scaffold samples, with a corresponding change in the perovskite nanocrystallite size in the mesoporous layer as the mesoporous scaffolds restrict the size, we confirm the trend of the emission peak with size for larger crystallites, well beyond the Bohr exciton radius, previously discussed for methylammonium lead iodide on the basis of experiments and simulations. Furthermore, the presence of very large crystals (Figure S11) with the emission maxima at longer wavelength (Figure S10) in the capping layer for the 95 nm mesoporous layer‐based sample compared to the capping layer of the 17 nm sample, is consistent with our previous conclusion in Figure S2 for methylammonium lead iodide demonstrating that the crystal size effect is present for quite large crystals in the capping layer as well and not limited to nanocrystallites in the mesoporous layer.

Secondly, these findings demonstrating the difference in opto‐electronic properties in terms of the band gap for perovskite in mesoporous scaffolds of different sizes, have practical implications as discussed in the previous section. These include improving the charge extraction in state‐of‐the‐art mesoscopic layer‐based solar cells and in the design of novel graded band gap devices constructed using mesoscopic layers to tune the band gap. Our work highlights the immense potential for designing perovskite opto‐electronic devices that exploit the phenomenon of crystal‐size‐induced band gap tuning conveniently via the use of tailored mesoscopic scaffolds.

In summary, the crystal size phenomenon we discussed is widely generalizable and we demonstrate that it influences various perovskites and deposition methods of current technological interest as well. The comparison of samples made with mesoporous layers of different particle sizes proves a) the effect of crystal size restriction on the opto‐electronic properties of the material and b) the practical use of this phenomenon in devices currently employing mesoscopic layers, as well as their potential for use in new designs.

## Conclusion

Our study demonstrates that methylammonium lead iodide perovskite films exhibit a blue‐shift in the PL spectra and the presence of an asymmetry in the emission during formation in the sequential deposition method, attributed to small perovskite nanocrystallites which are present at the early stages of the reaction. The asymmetry weighted towards higher energies is shown to be associated with the surface‐induced structural effects in small perovskite nanocrystallites, namely PbI_6_ octahedra tilting and Pb off‐centering. We distinguish this phenomenon from QC effects as changes in the perovskite band gap are observed for nanocrystallites of size greatly exceeding the Bohr exciton radius. We show that the perovskite in the mesoporous layer has a larger band gap compared to the capping layer due to the presence of smaller crystals in the former. Our results have broad implications for solar cells employing mesoporous architectures, as an intrinsic feature of the perovskite band gap for mesoporous layers, has been identified.

The wide scope of the crystal size effect is also demonstrated in our investigation of samples of the complex triple‐cation perovskite composition, prepared through the anti‐solvent method on mesoporous scaffolds of different particle sizes. This investigation confirms the ubiquitous nature and extensive applicability of the crystal size effect studied in our work in both: different perovskite compositions and various thin film deposition methods which are of current interest in the field. We show that band gap tuning can be conveniently achieved through control of the crystal size via the use of tailored mesoscopic scaffolds in perovskite solar cells. Overall, our work systematically unravels the phenomenon of crystal‐size‐induced band gap tuning in perovskites and highlights its immense potential for the design of highly efficient opto‐electronic devices in the near future.

## Conflict of interest

The authors declare no conflict of interest.

## Supporting information

As a service to our authors and readers, this journal provides supporting information supplied by the authors. Such materials are peer reviewed and may be re‐organized for online delivery, but are not copy‐edited or typeset. Technical support issues arising from supporting information (other than missing files) should be addressed to the authors.

Supporting Information
