# Supplementary material for: Crystal‐Size‐Induced Band Gap Tuning in Perovskite Films
Source: Angew Chem Int Ed Engl. 2021 Aug 18;60(39):21368–76. doi: 10.1002/anie.202106394 (PMC8518849; doi:10.1002/anie.202106394)
Supplement: Supplementary file 1 — Supporting Information [file ANIE-60-21368-s001.pdf]

Angewandte Chemie
Eine Zeitschrift der Gesellschaft Deutscher Chemiker

Supporting Information

Crystal-Size-Induced Band Gap Tuning in Perovskite Films

Amita Ummadisingu, Simone Meloni, Alessandro Mattoni, Wolfgang Tress, and Michael Grätzel**

anie_202106394_sm_miscellaneous_information.pdf

Author Contributions

A.U. conceptualized the study, conducted the PL and CLSM experiments and analyzed the data. A.U. and S.M. prepared the manuscript. S.M. planned the simulation campaign and performed the DFT calculations; A.M. conducted the classical MD simulations. W.T. participated in the analysis of results. M.G. directed the study. All authors discussed the results and commented on the manuscript.

Supporting Information

1. Materials and Methods

1.1. Materials

The materials used in the study were purchased either from Sigma-Aldrich or Acros Organics. MAI was prepared as reported in the report by Im et al.[1] The Al_2O_3 paste (23 nm diameter particles) was prepared in-house. Films used for the in-situ PL experiments were made using fluorine-doped tin oxide (FTO)-coated glass substrate (Tec15, Pilkington). Nippon sheet glass was used for the remaining experiments.

1.2. Sample preparation

Films used for experiments except for CLSM, had a mesoporous Al_2O_3 layer of 300 nm thickness. The mesoporous layer was deposited as mentioned in the report by Burschka et al.[2] For the samples used for CLSM imaging, a thick mesoporous Al_2O_3 layer of nearly 2.5 μm was deposited. Al_2O_3 mesoporous layers rather than those made of TiO_2 were employed in our samples to avoid quenching of the PL in both the steady state PL and CLSM measurements. To deposit the PbI_2 in the sequential deposition method, a 1.3 M solution of PbI_2 in N,N-dimethylformamide, kept at 70 °C, was spin coated at 6500 r.p.m. for 20 s. The sample was then heated at 70 °C for 10 min. We have infiltration of the PbI_2 into the mesoporous layer and the formation of a capping layer of PbI_2. A MAI solution in 2-propanol (6 mg ml^{-1}) was used in all experiments. Samples of specific reaction times were prepared by dipping PbI_2 films in MAI for the required period, followed by rinsing in 2-propanol to stop the reaction. They were then heated at 70 °C for 10 min. Fully converted samples of perovskite used for PL and XRD measurements have 300 nm of perovskite infiltrated into the mesoporous layer and an additional 300 nm of perovskite present as a capping layer. In comparison, samples fully converted to perovskite for CLSM have 2.5 μm of perovskite infiltrated into the thick mesoporous layer and a capping layer about 700 nm thick lying on top of it. The $Cs_{0.05}MA_{0.16}FA_{0.79}Pb(I_{0.83}Br_{0.17})_3$ perovskite samples were prepared on 1 to 1.5 μm thick Al_2O_3 mesoscopic layers composed of either 17 nm or 95 nm diameter particles, using the anti-solvent method as outlined in the literature[3]. Perovskite was infiltrated into the mesoporous layer and formed an additional capping layer for these samples.

1.3. Photoluminescence measurements

PL were carried out on a Horiba Jobin Yvon Fluorolog spectrofluorometer. For the steady state PL measurements conducted *ex-situ*, the excitation wavelength was 460 nm with a bandpass of 14 nm and the detection bandpass was 5 nm. In the *in-situ* PL measurements, the samples were placed vertically in a standard cuvette of 10-mm path length using a Teflon holder. First, pure 2-propanol was injected into the cuvette to wet the film. The solvent was then replaced by a solution of MAI in 2-propanol (6 mg ml^{-1}) and the photoluminescence was monitored after this point. Excitation wavelength was 450 nm with a bandpass of 5 nm and the detection bandpass was 5 nm.

1.4. Theory and Simulations

Classical MD: The simulated nanocrystallites were obtained aging initially cubic crystalline clusters for a total of ~150 ps at 300 K. The samples consisting of N x N x N (N=5-16) unit cells of the cubic structure were initially relaxed in periodic boundary conditions. Then, after creating large vacuum around the cubic structures, the clusters were gently warmed up to 300 K in 50 ps and thermalized for an additional 50 ps. Finally, structural properties were computed in a 300 K, 50 ps long run, during which we checked that pairs and angular distribution functions are well converged.

Model nanocrystals considered in this work contain both MAI and PbI_2 terminated surfaces. Literature is controversial concerning surface termination, with some articles supporting the hypothesis that MAX[4] (X=I, Br) termination is more stable and others that PbI_2 is more likely to be observed[5].

Haruyama et al.[5b] stress that the surface termination might depend on the preparation conditions of the perovskite film. Thus, given the complexity of the system considered, which concerns perovskites films along their formation, we modeled $CH_3NH_3PbI_3$ nanocrystals by a simplified model containing both types of terminations, which guaranteed the electroneutrality of the sample. Real nanocrystals can be significantly more complex, with defects at their surface or disorder (amorphization) beyond the one observed in our simulations. However, we remark that here we are only interested in the structural characteristics of the *bulk-like* core region of the nanocrystal, where PL emission takes place, and their dependence on its size. These structural characteristics are (indirectly) affected by the surface of the nanocrystal due to the stress induced by undercoordination of the atoms belonging to the top layer, which is present in both kinds of terminations.

Simulations were performed using the MYP1 force model[6], using the LAMMPS code[7]. Generally speaking, classical interaction potentials suffer from limited transferability, i.e., their use in conditions very different from those for which the potentials have been designed might hamper the accuracy of results. To limit this problem, some of the authors of the present work have kept improving the MYP potential over the years, producing new versions that are able to successfully model a broad range of systems. Limiting our attention to the case of surfaces, interfaces and nanograins, we mention that the MYP potential has successfully been employed to investigate the structure, energetics, and polarity of surfaces[6b] and interfaces[8], the interaction (trapping) between defects and surfaces[9] and grain boundaries[10], and mechanical and nanoductile behavior of (single crystal and) polycrystalline samples under mechanical loading[11].

DFT: Calculations have been performed using the same protocol the authors have employed in previous works[8a, 12]. In particular, we use the Generalized Gradient Approximation (GGA) to density functional theory in the Perdew–Burke–Ernzerhof (PBE) formulation[13]. The interaction between valence electrons, core electrons and nuclei were described by ultrasoft pseudopotentials. Kohn–Sham orbitals were expanded in a plane wave basis set with a kinetic energy cutoff of 40 Ry, and a cutoff of 320 Ry for the density. The Brillouin zone was sampled with a 4 x 4 x 4 Monkhorst–Pack grid of k-points[14]. We remark that this setup has been proven to accurately predict the band gap[15]. Moreover, here we are interested in the variation of the band gap with the atomistic structure rather than its absolute value. The above values are chosen by checking the convergence of total energy, band gap and atomic forces. Calculations were performed using the Quantum Espresso suite of codes[16].

Quantum confinement: The band gap shift due to QC was computed according to the Brus formula[17], $\Delta E = \hbar^2\pi^2/2\mu r^2 + 1.786/\varepsilon r$, where $\mu = (\mu_e^{-1} + \mu_h^{-1})^{-1}$ is the exciton reduced mass (μ_e and μ_h are the effective masses of electrons and holes, respectively), ε the dielectric constant and r the radius of the cluster. μ_e and μ_h have been determined in previous DFT calculations[12b, 18], while for ε we use the experimental value available in the literature[19].

Simulation strategy: benefits and drawbacks: To investigate the effect of the size of $MAPbI_3$ clusters on their electronic properties, one could, in principle, use only *ab initio* simulations: one could run *ab initio* (DFT) molecular dynamics and along the trajectory compute valence band maximum and conduction band minimum. However, the high computational cost of *ab initio* calculations makes this approach inadequate: one is limited to clusters of small sizes and short simulations, typically ~1000 atoms and ~3 ps, respectively[20]. With the increase of computational power, over the last few years the largest cluster one can simulate, and the duration of *ab initio* simulations has increased but one is still limited to values insufficient to address the questions analyzed in this work. It suffices to recall that the largest computational cluster considered in our classical molecular dynamic (MD) simulations, which shows characteristics still differing from bulk $MAPbI_3$, contains ~50000 atoms.

Thus, we decided to adopt a different strategy. We use extensive classical MD simulations to compute structural characteristics of clusters of large size, e.g., ‘tilting’ (Pb-I-Pb angle) and Pb Off-centering (distance between Pb and the center of the six I atoms of the corresponding octahedron), whose distribution for the various clusters are reported in figures 2b and 2c. ‘Bulk’ (periodic) samples with atomic configurations corresponding to the average value or maximum of the distributions of Pb-I-Pb angle and Pb off-centering are prepared to perform DFT calculations to investigate the effect of cluster size through the structural deformations on the electronic properties of perovskite nuclei (figures 2d and

2e). Among the others, this approach allows us to distinguish the effect of quantum confinement from structural changes of the ordered core of perovskite nuclei.

Aside from the benefits discussed above, our computational approach presents some ‘drawbacks’. Indeed, though much longer than *ab initio* simulations, the duration of classical molecular dynamics runs is still insufficient to completely sample the configurations of the disordered surface of clusters at room temperature. One could, in principle, address this problem by running multiple simulations starting from different initial conditions, e.g., starting from different initial velocities extracted from a Maxwell-Boltzmann distribution. However, this would not solve the problem as the overall duration of the simulations, the duration of one simulation multiplied by the number of simulations, hence their computational cost, exceeds the capacity of present supercomputers. The effect of the limited duration of classical MD and/or the limited number of independent simulations, is reflected in some uncertainty in the distributions $P(\alpha)$ and $P(r_{Pb})$. This is evident for the distributions of the 3.2 nm cluster in figures 2b and 2c but holds true also for the other clusters. The uncertainty in the structural data is reflected in a corresponding uncertainty in the electronic properties of the sample. For example, one notices an apparently abnormal value of the predicted PL maximum (figure 2d) and δE of the valence band maximum (figure 2e) for clusters of 5.8 nm. This uncertainty due to incomplete sampling of the constant temperature ensemble is, indeed, the computational analogues of the uncertainty of experimental data, the difference between data that one observes when performing an experimental measurement several times or on different samples supposedly equivalent. Typically, this uncertainty is not present in pure *ab initio* calculations, usually showing regular trends of the computed observables. This is because in *ab initio* calculations samples are typically obtained from geometry optimization or short *ab initio* molecular dynamics started from equivalent (among clusters of different size) initial configurations; in these cases, the system cannot explore other (meta)stable states. In the present work, these (meta)stable states are associated with surface defects, such as the one present at the top-left corner of the 10.1 nm cluster of figure 2a. The sampling of these (meta)stable states is important as, through the templating effect (see section 4.2 below), they affect the properties of the ordered core of perovskite nuclei.

In conclusion, the apparent drawback of irregular trends obtained from our hierarchical multiscale approach, based on the combination of classical and *ab initio* simulations, is the fingerprint of the complexity of the structural characteristics of halide perovskites clusters and nuclei. Our hierarchical multiscale approach allows to explore this complexity, though this exploration is still not exhaustive. Despite this limitation, we believe that our hierarchical multiscale approach is preferable with respect to pure *ab initio* molecular dynamics, which imposes even more severe limitations to the exploration of the configuration of clusters/nuclei.

1.5. CLSM and image processing

CLSM images were captured using the Leica Application Suite X software on a confocal laser scanning microscope (Leica TCS SP8), using a HC PL APO oil objective (63x/1.40). A 440 nm pulsed diode laser was used for excitation. The excitation power and the gain were chosen to optimize the dynamic range of the photomultiplier tube detector.

For figure 4a, single plane 512x512 images were acquired at 25 °C from a unidirectional scan of 600 Hz speed with line averaging of 4. The pinhole size was 1 Airy unit. The images were acquired at a resolution of 52 nm in xy and image bit depth was 8. For figures S10a and S10c, single plane 1024x1024 images were acquired.

Fiji was used for image processing. We collected the emission as several images (in steps of 5 nm each), giving us 32 images collectively capturing the emission between 640 and 800 nm. The summation of these 32 images was pseudo-colored and the color scale assignment has been shown in each of the figures 4a, S10a and S10c. The images have been cropped to show the area of interest. Furthermore, the emission was sampled in steps of 5 nm without any overlap in the wavelengths from one step to the next. Figures 4b, S10b and S10d indicate the central wavelength of each of these 5 nm steps as the X-coordinate corresponding to the normalized emission for that image.

1.6. X-ray diffraction measurements

The XRD measurements were carried out on an X'Pert MPD PRO from PANalytical operated in the Bragg-Brentano geometry. It has a ceramic tube (Cu anode, λ=1.54060 Å), a secondary graphite (002) monochromator and a RTMS X'Celerator detector. The automatic divergence slit and beam mask were adjusted based on the dimensions of the films. A step size of 0.008 ° and an acquisition time of up to 7.5 min deg^{-1} was chosen.

1.7. SEM Imaging

SEM images were captured on a high-resolution scanning electron microscope (Zeiss Merlin) equipped with the in-lens detector. Minor brightness and contrast adjustments, as well as the addition of scale bars was done using Fiji.

2. Photoluminescence measurements

2.1. Ex-situ measurements:

Here we present further analysis of the ex-situ steady state photoluminescence (PL) measurements presented in figure 1. A closer look at the spectra in figures 1a and 1b suggests that, in addition to the maxima shifting towards longer wavelengths with time, the emission is strongly asymmetric with a considerable tail towards higher energies at the beginning of the reaction, becoming more symmetric as the reaction proceeds. Through the analysis shown in figure 1c, we quantify the shift of the maxima towards longer wavelengths with dipping time. This is accompanied by a decrease in the full width at half maximum (FWHM) as seen in figure 1d. Simulations shown later will investigate this phenomenon in detail.

To understand deviations in the above-mentioned trends (in figures 1c and 1d) for data points of intermediate dipping times, we refer to our previous report[21] where we demonstrated that the conversion of the PbI_2 to perovskite in the mesoporous layer is delayed in time and occurs after the conversion of the capping layer. We attribute the deviations centered at 25 s to the formation of perovskite in the mesoporous layer. Further experimental investigation on the emission from the mesoporous layer is presented later.

The forming perovskite is found to be in the tetragonal phase on the basis of the emission maxima observed at the end of the formation process (at ~60 s for these samples[21]) and x-ray diffraction (XRD) measurements (see figure S3) which are in agreement with reference data at room temperature[22].

Morphological changes due to the phenomenon of Ostwald ripening take place at long dipping times as reported in the literature[21, 23] and perovskite crystals remain in the tetragonal phase. For samples of long dipping times of 200 and 400 s (figure 1b), which are beyond the point of complete conversion of PbI_2 to perovskite, there is no shift in the emission maxima (figure 1c) and no change in the FWHM (figure 1d). This can be partly attributed to the observation that the crystals in the film are large enough at these long dipping times that an additional increase in size due to Ostwald ripening does not affect these emission parameters.

2.2. In-situ measurements:

We monitor the *in-situ* formation of the perovskite through sequential deposition using PL spectroscopy. The PbI_2 films are deposited onto a mesoporous Al_2O_3 scaffold and then dipped in a MAI solution in 2-propanol to form a $CH_3NH_3PbI_3$ film. We measure the emission from the forming perovskite during the dipping stage as a function of time at different detection wavelengths (see *Materials and Methods* for details). The results, presented in figure S1a, show a shift in the emission profile to longer times, as detection wavelength gradually increases from 720 to 810 nm. This indicates that there is a red-shift with increasing dipping time, similar to our observations for the steady-state PL measurements of samples halted at different points of conversion (ex-situ, see figure 1). We monitor the amount of PbI_2 in the sample by recording the emission at a detection wavelength of 520 nm and we observe a decrease in the signal as it is consumed to form perovskite. We see that the end of the red-shift in the emission profile of perovskite with time is concurrent with the complete conversion of PbI_2 to perovskite indicated by the disappearance of the PbI_2 signal.

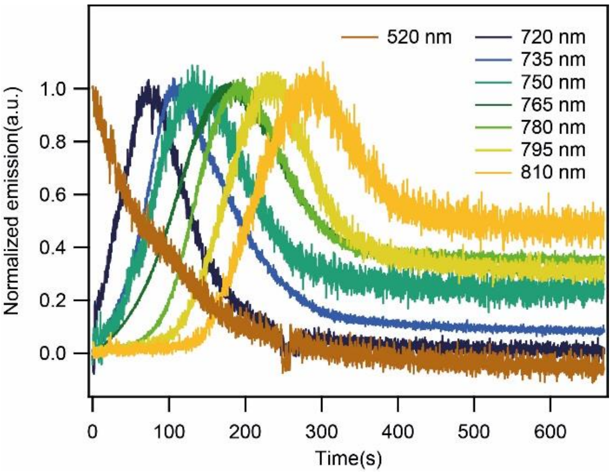

Figure S1: Normalized photoluminescence data as a function of time collected at different detection wavelengths monitoring perovskite formation in sequential deposition. *Detection wavelengths are indicated in the legend.*

2.3. Investigation of crystal size effect in capping layer:

Using the sequential deposition method, we prepare three samples with thin capping layers of $CH_3NH_3PbI_3$ perovskite on Al_2O_3 scaffolds. The crystals in the capping layer are of increasing size, while those in the mesoporous layer are considered identical as the same scaffold was used for all three samples. We conduct SEM and steady state PL measurements to investigate these samples and the results are shown in figure S2. We observe that smaller crystals in the capping layer also exhibit blue-shifted emission compared to larger crystals. This demonstrates that the phenomenon is therefore not limited to the nanocrystals in the mesoporous layer and it is omnipresent. This calls for a closer investigation, which is the objective of the current study.

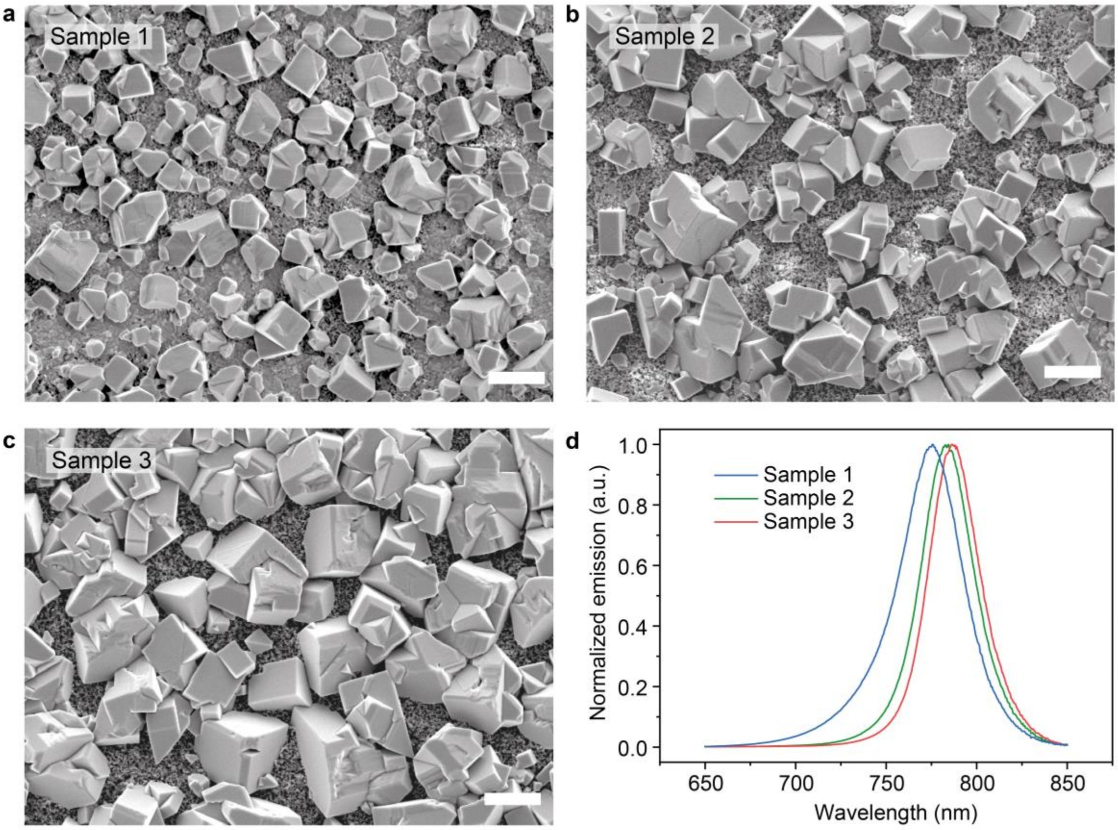

Figure S2: Investigation of $CH_3NH_3PbI_3$ perovskite films of different crystal sizes in the capping layer. *a-c) SEM images of three samples with increasing crystal sizes in the capping layer deposited*

on identical Al_2O_3 mesoporous layers underneath using the sequential deposition method. Scale bars, 1μm. d) Steady state PL spectra of samples shown in 'a-c'. Spectra show that smaller crystals in the capping layer also exhibit blue-shifted emission compared to larger crystals.

3. X-ray diffraction

Intermediate phases in the formation of perovskite are anticipated and they have been studied through techniques such as XRD as reported in the literature[24]. The intermediate DMF-based phases[24] if present in our samples would have been detected in the XRD spectra (figure S3) and we do not observe them. Furthermore, the steady-state PL measurements we perform (figure 1) show that the red-shift in PL is gradual and continuous as the perovskite formation reaction progresses indicating that it is likely due to a continuous phenomenon such as the growth of perovskite crystals in size rather than the presence of distinct intermediates. In case such intermediates were present, they are likely to be associated with distinct energy levels and we would expect to see distinct peaks in the emission profile as the perovskite forms and no such peaks are observed.

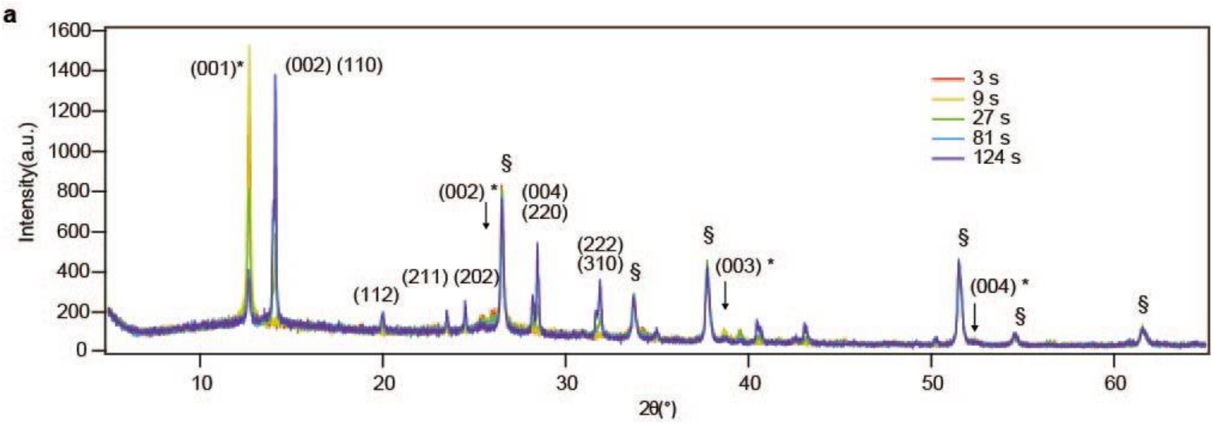

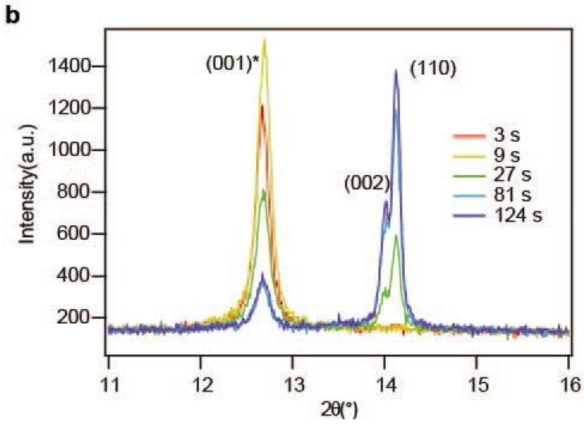

c

Dipping time(s)	FWHM(°)	Crystallite size(nm)
27	0.1020	77
81	0.0996	79
124	0.0975	81

Figure S3: XRD spectra and analysis of perovskite films dipped in MAI solution for different times. *a) Measured spectra and b) magnification of a part of the spectra. The main reflections corresponding to the tetragonal perovskite have been indicated. The reflections of the PbI_2 in the 2H polytype have been indicated with * and reflections from the FTO have been indicated with §. c) The full-width at half-maximum (FWHM) of the Voigt function fits of the (110) reflection of the tetragonal perovskite and the corresponding average crystallite size obtained using the Scherrer equation.*

4. Simulations

4.1. Molecular dynamics simulations:

In addition to the two hypotheses discussed in the main text, two other options are considered here and in the literature as possibilities responsible for the dependence of the PL spectrum on the configuration characteristics of perovskites. The first is that the size of $CH_3NH_3PbI_3$ nanocrystallite changes the distance between the Pb-I atoms forming the inorganic framework and through this the band gap[8a, 12a],

while the second is the rearrangement of the cation with the nanocrystallite size[22]. Figure S4 shows the Pb-I pair correlation function, namely g(r), measuring the distance of atoms in the inorganic framework, while figure S5 shows the distribution of the polar, p(ϕ), and azimuthal angles, p(θ), of the orientation of the C-N bond of $CH_3NH_3^+$ cations. We see that neither the g(r), nor the p(ϕ) and p(θ) show any significant change or correlation with the crystal size, apart from perhaps a minor sharpening of the g(r) with the nanocrystallite size.

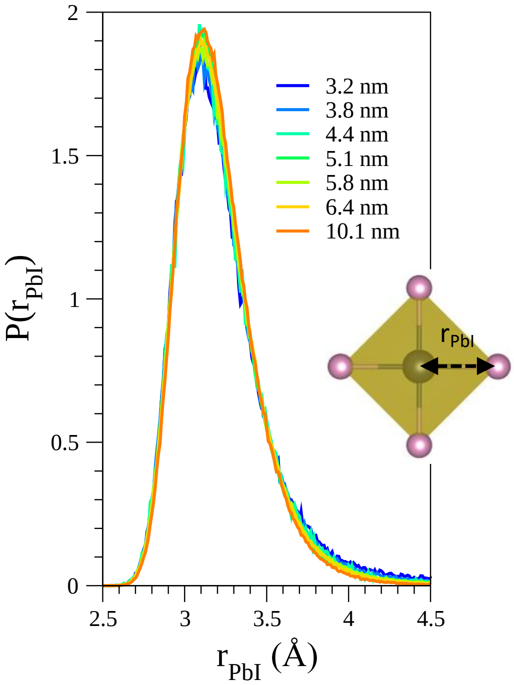

Figure S4: Pb-I distribution $P(r_{PbI})$. *No significant differences are observed in the positions of the peaks. The main difference is a tiny sharpening of the distribution.*

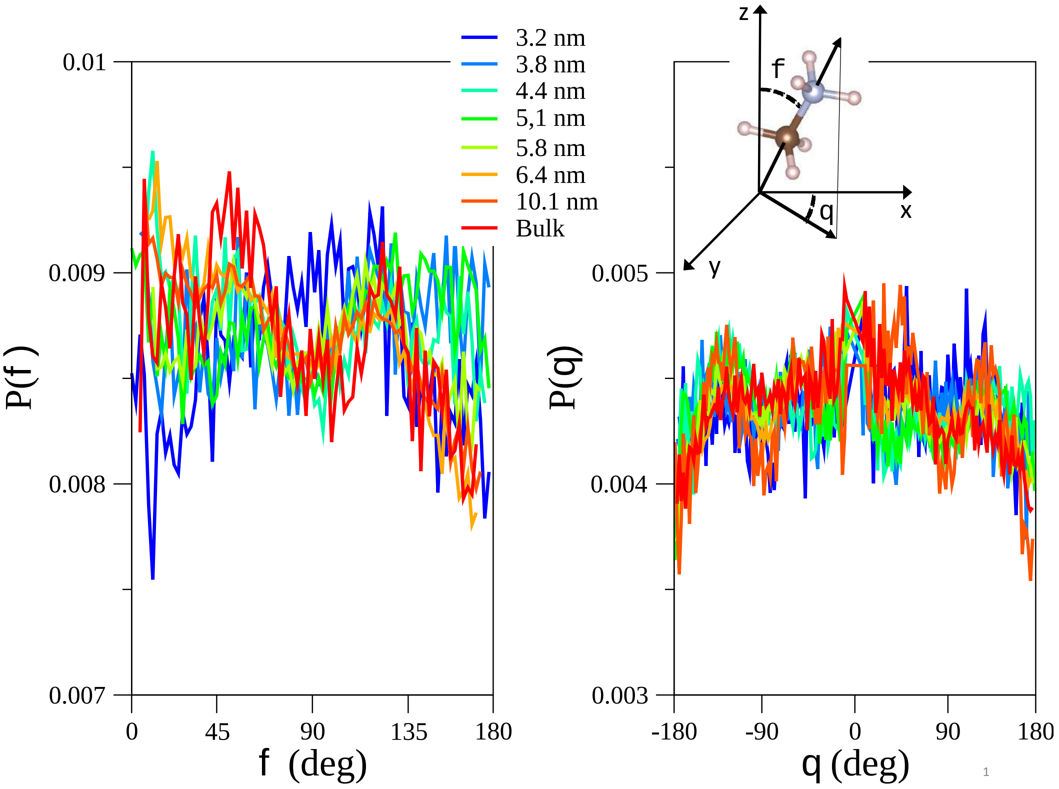

Figure S5: $CH_3NH_3^+$ orientation. *Orientation of the $CH_3NH_3^+$ cation as measured by the distribution of the polar (left) and azimuthal (right) angles of the C-N bonds in the various nanocrystallites and in the bulk. The rather flat shape of the distribution shows that, consistent with the literature[6a], at room temperature there are no significant preferential orientations of the cation but an almost quasi-random angular distribution. Both panels show that there is no clear dependence of the orientation of the cation among nanocrystallites and in comparison with the bulk.*

4.2. Templating effect of the nanocrystallite core:

In the main text, we discuss the templating effect of the core on the nanocrystallite periphery and the opposite disordering effect of the surface on the core. To substantiate this hypothesis, we compute the distribution of the atomic positions of the Pb and I atoms forming the inorganic 3D framework of $CH_3NH_3PbI_3$ nanocrystallites, $P(x)$. $P(x)$ along the (100) direction of three nanocrystallites of increasing size are reported in figure S6a, along with the corresponding distribution in an ideal (infinite) crystallite. $P(x)$ is characterized by an alternation of taller and shorter peaks, which are associated with layers containing one Pb and two I, and with layers containing only one I, respectively. In the bulk case (top line in the figure), the heights of the tall and short peaks change only marginally along the sample and these changes are due to the limited duration of the MD simulations. On the contrary, in the nanocrystallites, the peripheral peaks are broader and shorter, while the central ones are sharper and taller. This indicates that the center of the nanocrystallites is more bulk-like than the periphery, which unsurprisingly, is more disordered. Both the central and peripheral peaks show a sharpening with increase in the nanocrystallite size (bottom to top), indicating an increase in crystalline order in both regions. While the sharpening of the central peaks in the larger nanocrystallites is intuitive as the effect of structural disorder induced by the surface is expected to vanish after some crystal planes, the corresponding effect on peripheral peaks is somewhat surprising. We attribute this phenomenon to the *templating effect* of the more ordered core in larger nanocrystallites. This is also reflected in the Pb-I-Pb angles. In figure S6b, we report the distribution $P(\alpha)$ computed in cubic shell at various distances from the surface. In figure S6c, we show the maximum of these distributions for each nanocrystallite. As expected, α_{max} increases as we move from the surface towards the center of the nanocrystallites. However, we also notice that the α_{max} at a prescribed distance from the surface grows with the nanocrystallite size. This happens for both the core and the periphery of the nanocrystallite. This confirms that the templating effect mentioned in the main text and described here in more detail also affects the Pb-I-Pb angle and the off-centering, the two quantities we identify as primarily responsible for the change in PL spectra with nanocrystallite size.

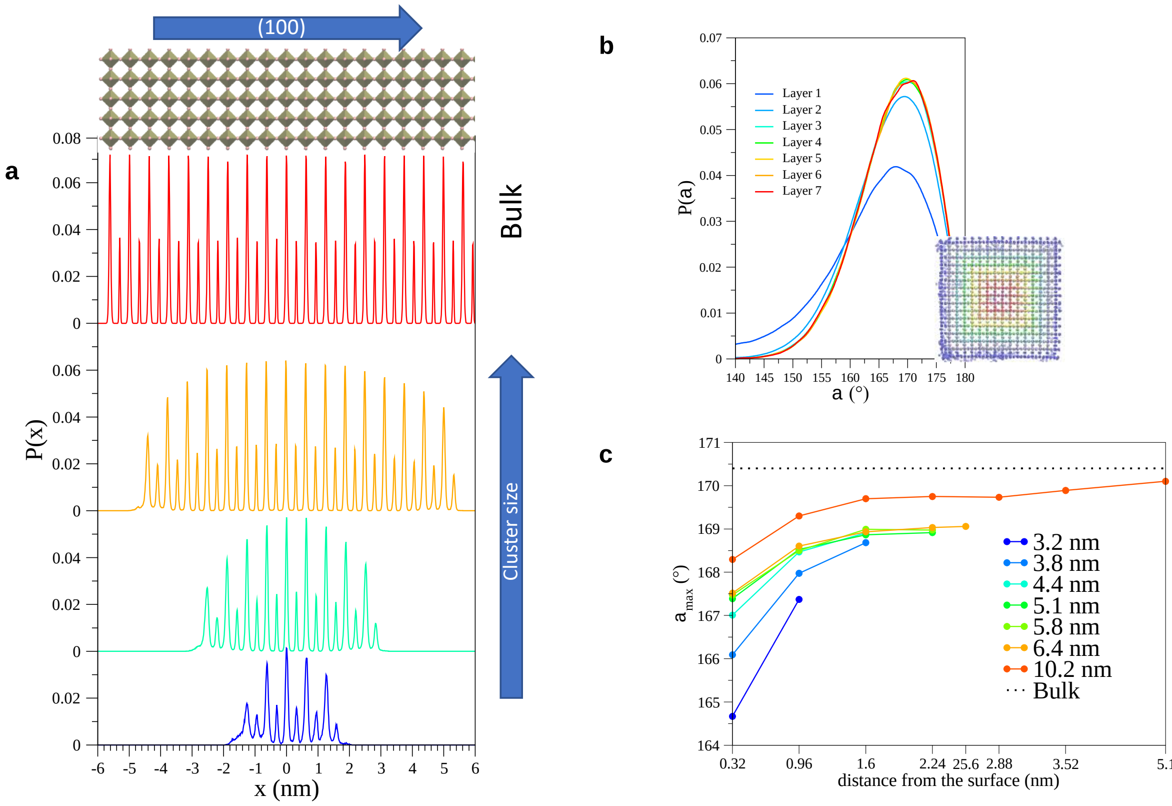

Figure S6: Distribution of the Pb and I atomic position along the (100) direction, $P(r_{PbI})$, and angle distribution shell-by-shell. *a) The distribution $P(r_{PbI})$ for nanocrystallites of increasing size (bottom to top) shown along with the corresponding distribution for an ideal (infinite) crystal. The figure shows that larger nanocrystallites present a distribution approaching the bulk one. This is reflected in the Pb-I-Pb distribution. b) Panel shows the Pb-I-Pb angle distribution discussed in the main text, computed in shells of the nanocrystallite at a prescribed distance from the surface, $P(\alpha)$. In particular, here we report the shell-by-shell $P(\alpha)$ for the 10.2 nm nanocrystallite. c) Maximum of the shell-by-shell $P(\alpha)$ for the different nanocrystallites.*

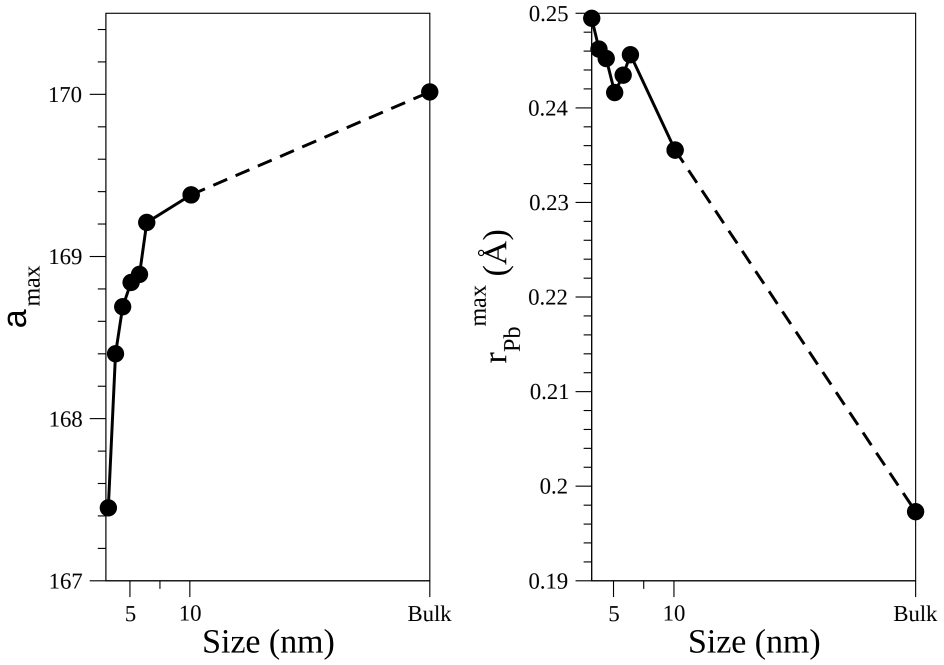

Figure S7: $\boldsymbol{\alpha_{max}}$ and $\mathbf{r_{Pb}}^{\mathbf{max}}$. *$\alpha_{max}$ (left) and r_{Pb}^{max} (right), the maxima of the distributions $P(\alpha_{\max})$ and $P(r_{Pb}^{max})$ of figure 2b and 2c, are the states of maximum probability of the system with respect to these two structural characteristics. These values have been computed by fitting the distribution of figure 2 around the maximum with a parabolic curve.*

5. SEM and BET analysis

SEM images of the surface of mesoporous films composed of 18 NRT TiO_2 and 30 NRD TiO_2 nanoparticles deposited on FTO glass are shown in figure S8. The nanoparticle size and pore size of each of these mesoporous layers can be visualized through these images with the 18 NRT TiO_2 clearly consisting of smaller nanoparticles as expected.

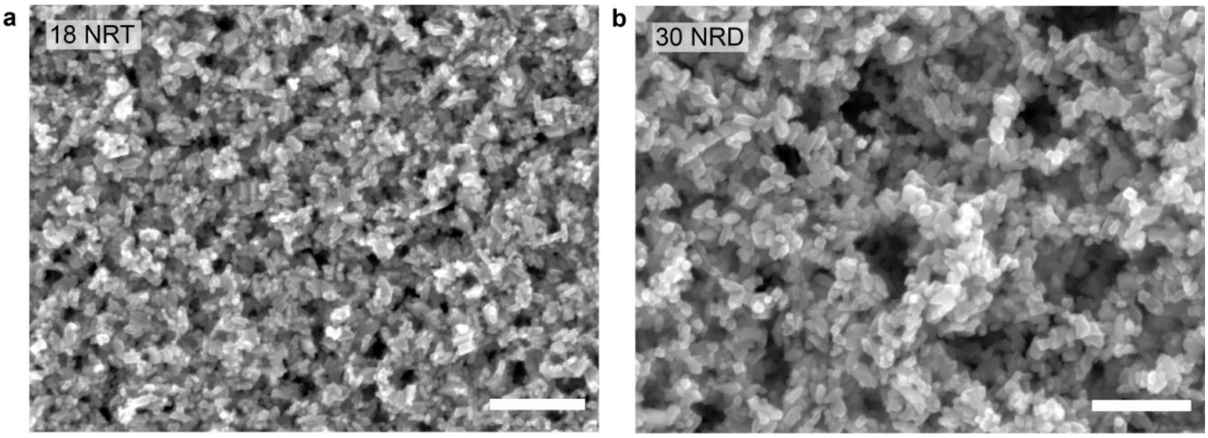

Figure S8: SEM images of the surface of mesoporous layers *a) 18 NRT TiO_2, b) 30 NRD TiO_2. Scale bars (a, b) 200 nm.*

In order to further characterize these mesoporous layers, we turn to N_2 gas adsorption measurements. We estimate the surface area and mesopore size distribution from the isotherms using the Brunauer-Emmett-Teller (BET) and Barrett-Joyner-Halenda (BJH) methods[25]. Table S1 summarizes the results of the BET and BJH analysis of 18 NRT TiO_2 and 30 NRD TiO_2 nanoparticles. From the BET analysis we obtain surface areas of 84.83 and 67.62 m^2g^{-1} respectively and average pore diameters (4V/A by BET) of approximately 25.89 and 30.81 nm respectively. The BJH analysis for desorption gives average pore diameters (4V/A) of 21.74 and 27.09 nm for 18 NRT TiO_2 and 30 NRD TiO_2 nanoparticles respectively.

Table S1: Summary of the BET and BJH analysis of mesoporous particles *a) 18 NRT TiO_2, b) 30 NRD TiO_2.*

Method	Parameter (units)	18 NRT	30 NRD
BET	Surface area ($m^2 g^{-1}$)	84.83	67.62
	Average pore diameter (nm)	25.89	30.81
BJH (Desorption)	Cumulative surface area between 1 and 500 nm diameter of pores ($m^2 g^{-1}$)	99.11	77.76
	Cumulative pore volume between 1 and 500 nm diameter of pores ($cm^2 g^{-1}$)	0.54	0.53
	Average pore diameter (nm)	21.74	27.09

6. Broader context for solar cell design

Figure S9 shows the proposed perovskite solar cell design with the perovskite in the various mesoporous layers of different particle sizes and the capping layer. To tune the band gap to be beneficial for such solar cells, the mesoporous layers could be deposited in such a way that the smallest mesoporous particles layer, associated with the largest band gap of the perovskite nanocrystallites infiltrated in it, is in contact with the electrode (in this case, the FTO substrate). Increasingly larger mesoscopic particle sizes that give smaller band gaps for perovskite nanocrystallites in them could be used for the subsequent layers moving towards the capping layer.

We also considered the possibility of re-absorption of the perovskite emission in the proposed solar cell design. This has been studied in the past by Pazos-Outón et al.[26] in their work on photon recycling in perovskites where they report that the internal photon spectrum peak shifts from 765 nm towards the red to ≥800 nm as a result of repeated photon re-absorption and emission in the thin film. In our proposed graded solar cell composed of various mesoscopic layers, the infiltrated perovskite nanocrystallites would have larger band gaps and blue-shifted emissions compared to the capping layer, as shown schematically in Figure S9. As the phenomenon reported by Pazos-Outón et al.[26] results in large red-shifts not blue-shifts, it is likely to be present in the graded solar cell. Moreover, they report that the phenomenon is beneficial for solar cells and results in high excitation densities in the perovskite layer and facilitates high open-circuit voltages[26].

Pazos-Outón et al.[26] have also demonstrated that through this photon recycling phenomenon, charge extraction for excitation at long distances of more than 50 μm from the contacts was feasible in lateral-contact solar cells, indicating that energy transport in perovskite thin films is not limited by diffusive charge transport on these length scales. It is probable that the same phenomenon would be present in the proposed graded solar cells and charge transport is not a bottleneck given the total thickness of the perovskite layers would be at least an order of magnitude smaller than 50 μm.

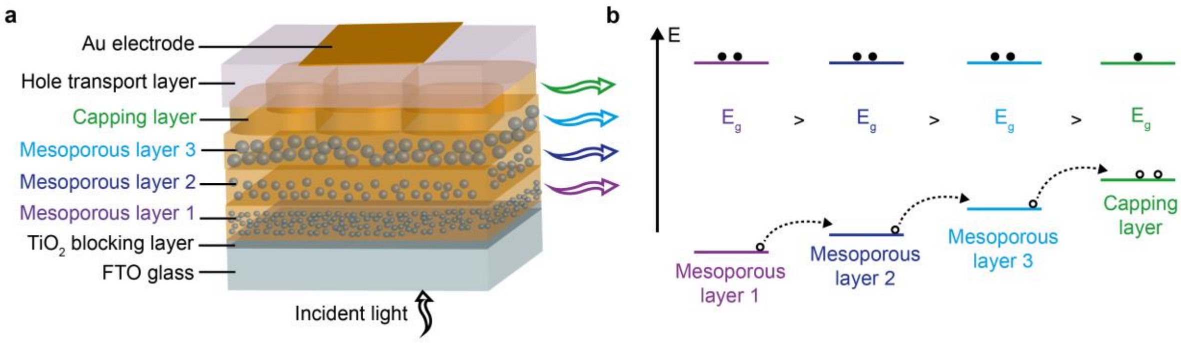

Figure S9: Schematics showing the perovskite in the various mesoporous layers of different particle sizes and the capping layer in the proposed perovskite solar cell design. *a) View of the solar cell depicted with the emission from different layers b) energy band diagram depicting the perovskite levels in the different mesoporous and capping layers.*

7. CLSM and SEM analysis

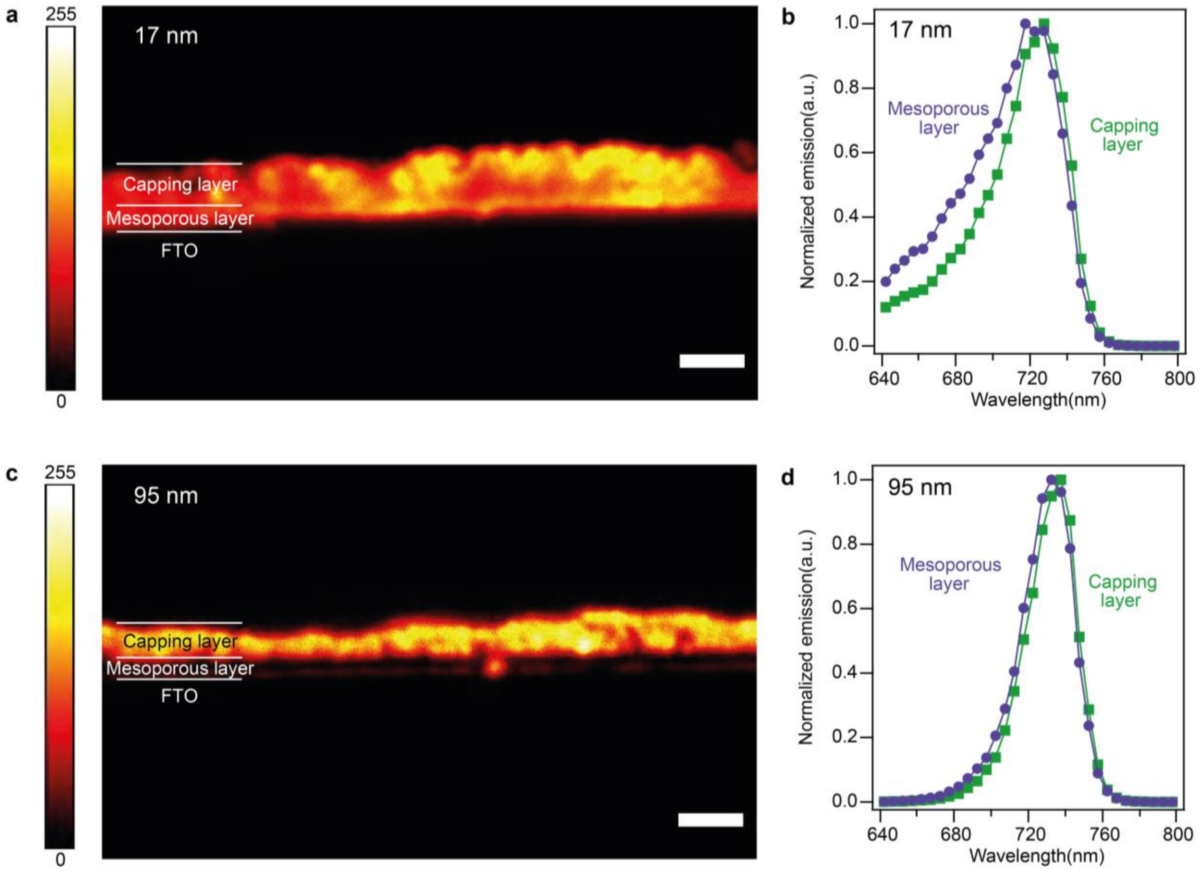

Figure S10: Study of the emission from the mesoporous layer and the capping layer in a partial cell. *a, b) Sample made using 17 nm diameter Al_2O_3 particles in its mesoporous layer. c, d) Sample made using 95 nm diameter Al_2O_3 particles in its mesoporous layer. a, c) Cross-sectional CLSM imaging of $Cs_{0.05}MA_{0.16}FA_{0.79}Pb(I_{0.83}Br_{0.17})_3$ sample consisting of a capping layer and the perovskite infiltrated into an Al_2O_3 mesoporous scaffold. Pseudo-color image shown. 32 images showing the emission between 640 and 800 nm in steps of 5 nm each have been summed. Color scale assignment to the emission intensity in the images is shown. Scale bars, 2.5 μm. b, d) Normalized emission from the perovskite in the capping layer and the mesoporous layer obtained from the images that make up 'a' and 'c' respectively. The perovskite emission from the mesoporous layer is blue-shifted compared to the capping layer.*

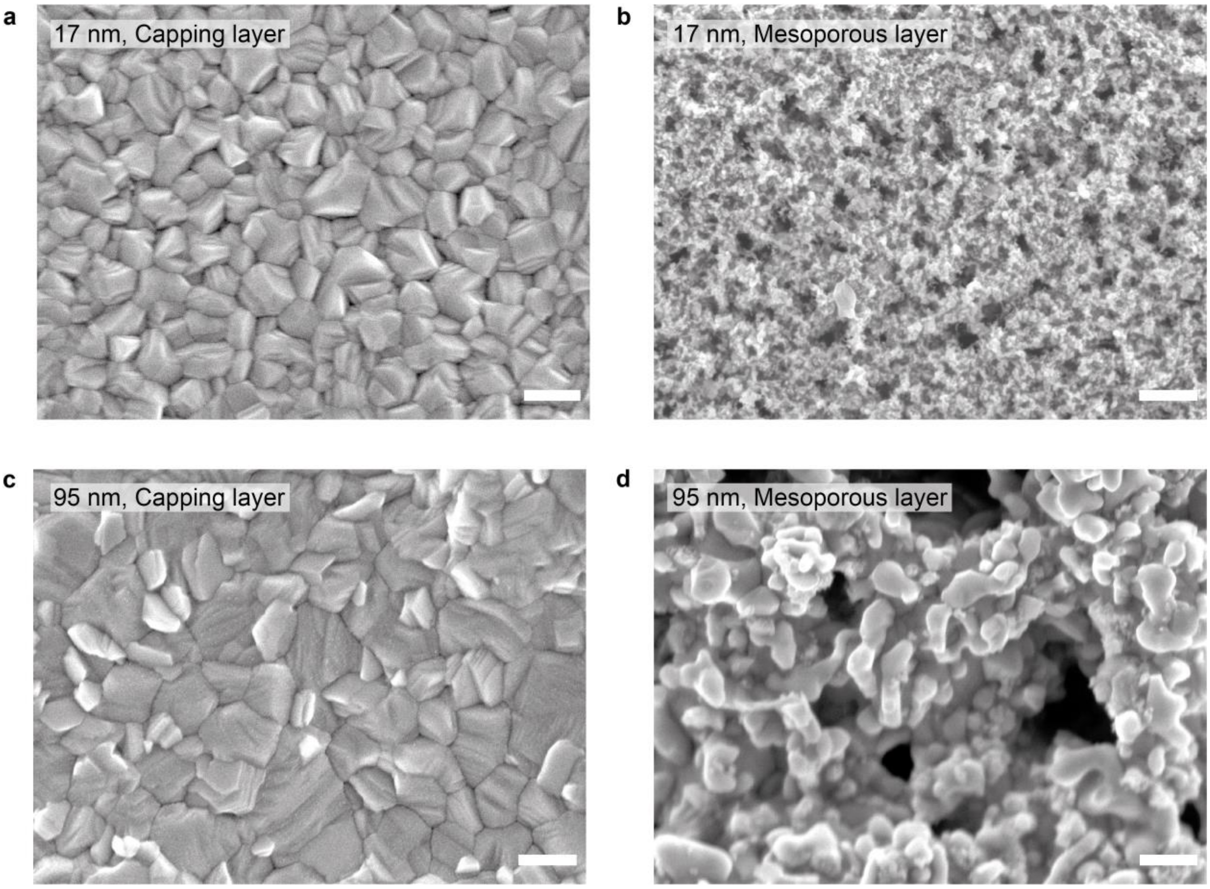

Figure S11: SEM investigation of the morphology of the mesoporous layer and the capping layer of the $Cs_{0.05}MA_{0.16}FA_{0.79}Pb(I_{0.83}Br_{0.17})_3$ samples shown in figure S10. Mesoporous layers are infiltrated with the perovskite. *a, b) Sample made using 17 nm diameter Al_2O_3 particles in its mesoporous layer. c, d) Sample made using 95 nm diameter Al_2O_3 particles in its mesoporous layer. a, c) Capping layer, b, d) mesoporous layer. Scale bars, 300 nm.*

References

[1] J. H. Im, C. R. Lee, J. W. Lee, S. W. Park, N. G. Park, *Nanoscale* **2011**, *3*, 4088-4093.

[2] J. Burschka, N. Pellet, S. J. Moon, R. Humphry-Baker, P. Gao, M. K. Nazeeruddin, M. Grätzel, *Nature* **2013**, *499*, 316-319.

[3] M. Saliba, T. Matsui, J. Y. Seo, K. Domanski, J. P. Correa-Baena, M. K. Nazeeruddin, S. M. Zakeeruddin, W. Tress, A. Abate, A. Hagfeldt, M. Grätzel, *Energy Environ. Sci.* **2016**, *9*, 1989-1997.

[4] a) L. She, M. Liu, D. Zhong, *ACS nano* **2016**, *10*, 1126-1131; b) X. Huang, T. R. Paudel, P. A. Dowben, S. Dong, E. Y. Tsymbal, *Phys. Rev. B* **2016**, *94*, 195309; c) T. Komesu, X. Huang, T. R. Paudel, Y. B. Losovyj, X. Zhang, E. F. Schwier, Y. Kojima, M. Zheng, H. Iwasawa, K. Shimada, M. I. Saidaminov, D. Shi, A. L. Abdelhady, O. M. Bakr, S. Dong, E. Y. Tsymbal, P. A. Dowben, *J. Phys. Chem. C* **2016**, *120*, 21710-21715; d) S. Masi, F. Aiello, A. Listorti, F. Balzano, D. Altamura, C. Giannini, R. Caliandro, G. Uccello-Barretta, A. Rizzo, S. Colella, *Chem. Sci.* **2018**, *9*, 3200-3208.

[5] a) E. Mosconi, J. M. Azpiroz, F. De Angelis, *Chem. Mater.* **2015**, *27*, 4885-4892; b) J. Haruyama, K. Sodeyama, L. Han, Y. Tateyama, *J. Phys. Chem. Lett.* **2014**, *5*, 2903-2909; c) Y. Wang, B. G. Sumpter, J. Huang, H. Zhang, P. Liu, H. Yang, H. Zhao, *J. Phys. Chem. C* **2015**, *119*, 1136-1145.

[6] a) A. Mattoni, A. Filippetti, M. I. Saba, P. Delugas, *J. Phys. Chem. C* **2015**, *119*, 17421-17428; b) C. Caddeo, M. I. Saba, S. Meloni, A. Filippetti, A. Mattoni, *ACS Nano* **2017**, *11*, 9183-9190.

[7] S. Plimpton, *J. Comput. Phys.* **1995**, *117*, 1-19.

[8] a) M. I. Dar, G. Jacopin, S. Meloni, A. Mattoni, N. Arora, A. Boziki, S. M. Zakeeruddin, U. Rothlisberger, M. Grätzel, *Sci. Adv.* **2016**, *2*, e1601156; b) C. Caddeo, D. Marongiu, S. Meloni, A. Filippetti, F. Quochi, M. Saba, A. Mattoni, *Adv. Mater. Interfaces* **2018**, *6*, 1801173.

[9] C. Caddeo, A. Filippetti, A. Mattoni, *Nano Energy* **2020**, *67*, 104162.

[10] N. Phung, A. Al-Ashouri, S. Meloni, A. Mattoni, S. Albrecht, E. L. Unger, A. Merdasa, A. Abate, *Adv. Energy Mater.* **2020**, *10*, 1903735.

[11] J. Yu, M. Wang, S. Lin, *ACS Nano* **2016**, *10*, 11044-11057.

[12] a) S. Meloni, G. Palermo, N. Ashari-Astani, M. Grätzel, U. Rothlisberger, *J. Mater. Chem. A* **2016**, *4*, 15997-16002; b) N. Ashari-Astani, S. Meloni, A. H. Salavati, G. Palermo, M. Grätzel, U. Rothlisberger, *J. Phys. Chem. C* **2017**, *121*, 23886-23895; c) C. Yi, J. Luo, S. Meloni, A. Boziki, N. Ashari-Astani, C. Grätzel, S. M. Zakeeruddin, U. Röthlisberger, M. Grätzel, *Energy Environ. Sci.* **2016**, *9*, 656-662; d) S. Meloni, T. Moehl, W. Tress, M. Franckevičius, M. Saliba, Y. H. Lee, P. Gao, M. K. Nazeeruddin, S. M. Zakeeruddin, U. Rothlisberger, M. Grätzel, *Nat. Commun.* **2016**, *7*, 10334.

[13] J. P. Perdew, K. Burke, Y. Wang, *Phys. Rev. B Condens. Matter* **1996**, *54*, 16533-16539.

[14] H. J. Monkhorst, J. D. Pack, *Phys. Rev. B* **1976**, *13*, 5188-5192.

[15] P. Umari, E. Mosconi, F. De Angelis, *Sci. Rep.* **2014**, *4*, 4467.

[16] G. Paolo, B. Stefano, B. Nicola, C. Matteo, C. Roberto, C. Carlo, C. Davide, L. C. Guido, C. Matteo, D. Ismaila, C. Andrea Dal, G. Stefano de, F. Stefano, F. Guido, G. Ralph, G. Uwe, G. Christos, K. Anton, L. Michele, M.-S. Layla, M. Nicola, M. Francesco, M. Riccardo, P. Stefano, P. Alfredo, P. Lorenzo, S. Carlo, S. Sandro, S. Gabriele, P. S. Ari, S. Alexander, U. Paolo, M. W. Renata, *J. Phys. Condens. Matter* **2009**, *21*, 395502.

[17] L. E. Brus, *J. Chem. Phys.* **1984**, *80*, 4403-4409.

[18] J. Feng, B. Xiao, *J. Phys. Chem. Lett.* **2014**, *5*, 1278-1282.

[19] M. Samiee, S. Konduri, B. Ganapathy, R. Kottokkaran, H. A. Abbas, A. Kitahara, P. Joshi, L. Zhang, M. Noack, V. Dalal, *Appl. Phys. Lett.* **2014**, *105*, 153502.

[20] G. Giorgi, K. Yamashita, *J. Phys. Chem. Lett.* **2016**, *7*, 888-899.

[21] A. Ummadisingu, M. Grätzel, *Sci. Adv.* **2018**, *4*, e1701402.

[22] a) G. Grancini, S. Marras, M. Prato, C. Giannini, C. Quarti, F. De Angelis, M. De Bastiani, G. E. Eperon, H. J. Snaith, L. Manna, A. Petrozza, *J. Phys. Chem. Lett.* **2014**, *5*, 3836-3842; b) T. J. Jacobsson, L. J. Schwan, M. Ottosson, A. Hagfeldt, T. Edvinsson, *Inorg. Chem.* **2015**, *54*, 10678-10685.

[23] a) D. H. Cao, C. C. Stoumpos, C. D. Malliakas, M. J. Katz, O. K. Farha, J. T. Hupp, M. G. Kanatzidis, *APL Mater.* **2014**, *2*, 091101; b) A. Ummadisingu, L. Steier, J. Y. Seo, T. Matsui, A. Abate, W. Tress, M. Grätzel, *Nature* **2017**, *545*, 208-212.

[24] A. A. Petrov, I. P. Sokolova, N. A. Belich, G. S. Peters, P. V. Dorovatovskii, Y. V. Zubavichus, V. N. Khrustalev, A. V. Petrov, M. Grätzel, E. A. Goodilin, A. B. Tarasov, *J. Phys. Chem. C* **2017**, *121*, 20739-20743.

[25] J. Rouquérol, D. Avnir, C. W. Fairbridge, D. H. Everett, J. M. Haynes, N. Pernicone, J. Ramsay, K. S. W. Sing, K. K. Ünger, *Pure Appl. Chem.* **1994**, *66*, 1739-1758.

[26] L. M. Pazos-Outón, M. Szumilo, R. Lamboll, J. M. Richter, M. Crespo-Quesada, M. Abdi-Jalebi, H. J. Beeson, M. Vrućinić, M. Alsari, H. J. Snaith, B. Ehrler, R. H. Friend, F. Deschler, *Science* **2016**, 351, 1430-1433.
